# Inhibition of lactate transport by MCT-1 blockade improves chimeric antigen receptor T-cell therapy against B-cell malignancies

**DOI:** 10.1136/jitc-2022-006287

**Published:** 2023-06-30

**Authors:** Ernesto Lopez, Rajesh Karattil, Francesco Nannini, Gordon Weng-Kit Cheung, Lilian Denzler, Felipe Galvez-Cancino, Sergio Quezada, Martin A Pule

**Affiliations:** 1 Haematology Department, Cancer Institute, University College London, London, UK; 2 Cancer Immunology Unit, Cancer Institute, University College London, London, UK; 3 Division of Biosciences, Institute of Structural and Molecular Biology, University College London, London, UK

**Keywords:** Immunotherapy, Metabolic Networks and Pathways, Receptors, Chimeric Antigen, T-Lymphocytes

## Abstract

**Background:**

Chimeric antigen receptor (CAR) T cells have shown remarkable results against B-cell malignancies, but only a minority of patients have long-term remission. The metabolic requirements of both tumor cells and activated T cells result in production of lactate. The export of lactate is facilitated by expression of monocarboxylate transporter (MCTs). CAR T cells express high levels of MCT-1 and MCT-4 on activation, while certain tumors predominantly express MCT-1.

**Methods:**

Here, we studied the combination of CD19-specific CAR T-cell therapy with pharmacological blockade of MCT-1 against B-cell lymphoma.

**Results:**

MCT-1 inhibition with small molecules AZD3965 or AR-C155858 induced CAR T-cell metabolic rewiring but their effector function and phenotype remained unchanged, suggesting CAR T cells are insensitive to MCT-1 inhibition. Moreover, improved cytotoxicity in vitro and antitumoral control on mouse models was found with the combination of CAR T cells and MCT-1 blockade.

**Conclusion:**

This work highlights the potential of selective targeting of lactate metabolism via MCT-1 in combination with CAR T cells therapies against B-cell malignancies.

**WHAT IS ALREADY KNOWN ON THIS TOPIC**

- Chimeric antigen receptor (CAR) T cells have proven highly effective in treating refractory lymphoid malignancies. However, most patients either subsequently relapse or do not respond.

**WHAT THIS STUDY ADDS**

- By exploiting metabolic vulnerabilities of B-cell cancer cells, we increased the antitumoral potential of CD19-specific CAR T cells via combination with pharmacological blockade of the lactate exporter monocarboxylate transporter-1.

**HOW THIS STUDY MIGHT AFFECT RESEARCH, PRACTICE OR POLICY**

- Our findings support the development of new antitumoral treatments by combining lactate modulators and CAR T cells in patients with B-cell malignancies.

## Background

Adoptive cell therapy (ACT) with chimeric antigen receptors (CARs) engineered T cells has proven highly effective in treating refractory lymphoid malignancies.^1–3^ Despite long-term remissions in a proportion of patients with B-cell malignancies, most either subsequently relapse or do not respond.^4 5^ While some relapses are due to antigen escape, most occur in the presence of cognate antigen. Furthermore, CAR T-cell therapy has had limited success in the treatment of non-lymphoid malignancy.^6 7^ These observations indicate that strategies to improve CAR T-cell efficacy are needed. Development of antitumor therapies which complement or synergize CAR T cells is a potential strategy which can improve outcomes.

Both tumor and cytotoxic T cells are highly proliferative and engage in similar metabolic networks, relying on glucose uptake and lactate fermentation to sustain their energetic demands.^8^ Moreover, diminishing glycolytic engagement on tumor cells improved the therapeutic potential of several immunotherapies.^9^ Modulating glycolytic activity of CAR T cells showed increased memory T-cell formation and enhanced the therapeutic potential of CAR T cells in several preclinical models.^10^ Furthermore, CAR T cells with a 4-1BB endodomain have increased mitochondria biogenesis, preferentially engaged in oxidative phosphorylation favoring T-cell persistence and accumulation of memory CAR T cells.^11^ Thus, several strategies have been developed to exploit tumor metabolic requirements while improving CAR T-cell therapies.

Monocarboxylate transporters (MCT) are a family of proton-link plasma membrane transporters and are the main exporters of lactic acid to the tumor microenvironment (TME). T cells upregulate MCT-1, MCT-2 and MCT-4 on activation^12^ while MCT-1 and MCT-4 are the most common isoforms in cancer, and their expression is correlated with worse prognosis in several malignancies.^13^ High concentrations of lactic acid in TME directly impair T-cell responses by inducing reductive stress, diminishing proliferation and cytokine production of T cells.^14 15^ Systemic administration of small molecules blocking lactate export through MCT-1 impaired tumor growth in immunodeficient mice models of melanoma, B-cell lymphoma, breast carcinoma and lung carcinoma, among others,^16 17^ and their efficacy is being explored in clinical trials against solid tumors (NCT01791595).^18^ Furthermore, MCT-1 inhibition increased the efficacy of anti-programmed cell death protein 1(αPD-1) immune checkpoint inhibition against solid tumors,^19 20^ reduced the intratumoral concentration of lactic acid^21^ and increased immune infiltration,^17^ demonstrating the potential synergy with other immunotherapies.

Blocking MCT-1/2 on phorbol myristate acetate (PMA)/ionomycin stimulated T cells showed less glycolytic engagement, impaired proliferation, and reduced effector function.^14 22^ In contrast, pharmacological inhibition of MCT-1 or dual blockade of MCT-1/4 with diclofenac, a non-steroidal anti-inflammatory drug, reduced lactate secretion and diminished glucose uptake without compromising T-cell function,^23^ suggesting comprehensive studies must be performed in the context of each immunotherapy. Taken together, glycolytic and lactate regulation represents an exciting approach for combination with T-cell immunotherapies, particularly on CAR T cells.

Here, we explored the combination of CD19-specific CAR T cells with pharmacological blockade of MCT-1. B-cell lymphomas predominately use MCT-1 for lactate export, and are sensitive to inhibition, but have little or no expression of MCT-4.^24 25^ In contrast, CAR T cells should express MCT-4. We hence hypothesized that CAR T-cell therapy would be synergistic with MCT-1 blockade when targeting B-cell lymphoma. We found that CAR T cells expressed high levels of both MCT-1 and MCT-4 on activation and perhaps consequently, they were functionally insensitive to MCT-1 inhibition with small molecules AZD3965 or AR-C155858. Combination of CAR T cells and MCT-1 blockade improved cytotoxicity in vitro and tumor clearance in vivo. This work highlights the potential of targeting lactate metabolism via MCT-1 in combination with CAR T-cell therapies against B-cell malignancies.

## Results

### Expression and function of MCT on CAR T cells

**Figure 1:**
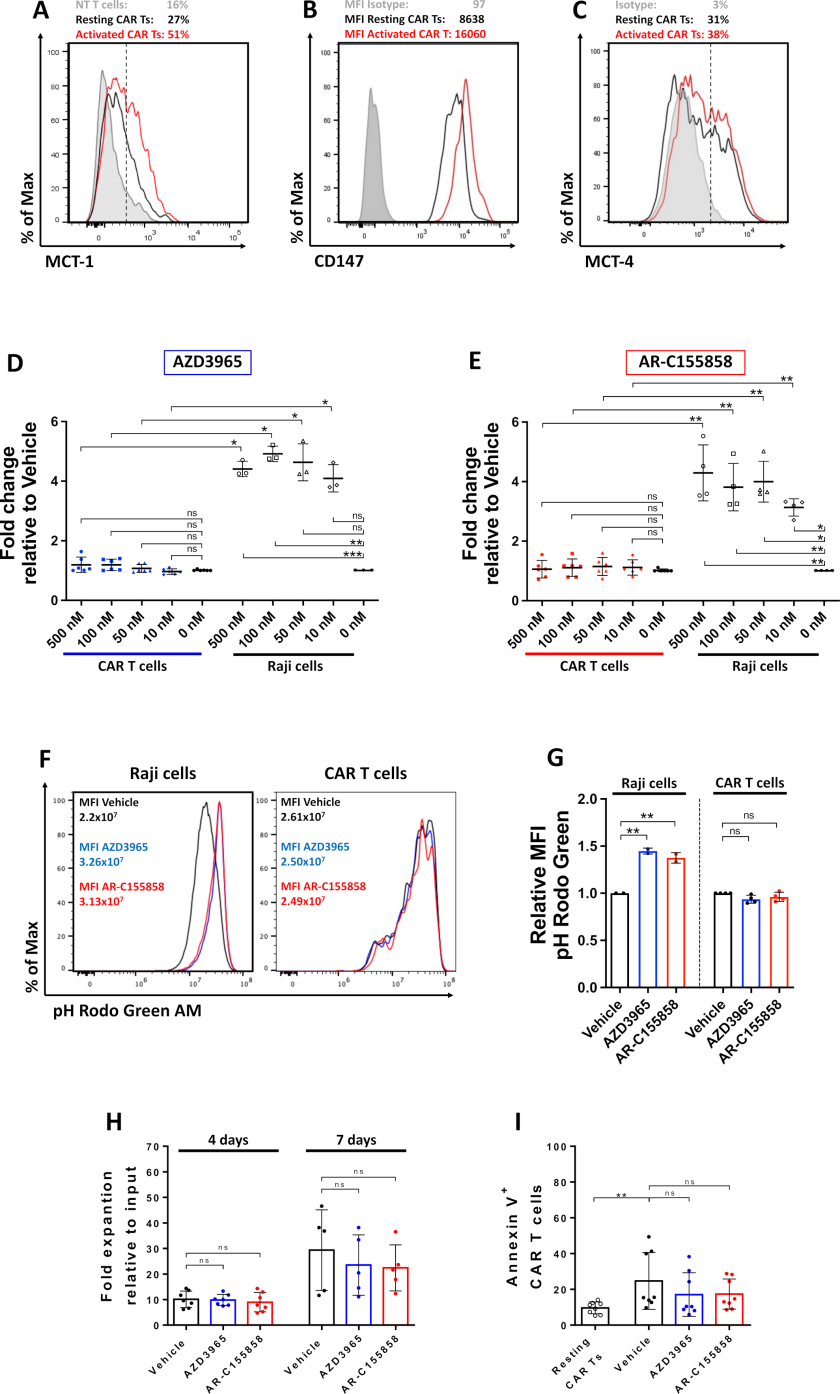
Expression and functionality of MCTs on CAR T cells. Non-transduced or αCD19-CAR T cells were cultured with Raji or Raji-CD19^KO^ cells for 24 hours. Representative histogram of (A) MCT-1, (B) CD147 and (C) MCT-4 expression on CAR T cells measured by flow cytometry. Intracellular lactate relative to vehicle control on cells cultured with (D) AZD3965 or (E) AR-C155858. (F) Representative histogram and (G) relative MFI of pH Rodo Green AM in Raji cells and activated CAR T cells after 48 hours of activation. (H) Fold expansion of CAR T cells after 4 and 7 days of culture with 100 nM of MCT-1 inhibitors. (I) Expression of annexin V on CAR T cells after 48 hours of activation measured by flow cytometry. Pooled data of two or three independent experiments, n=4–7 healthy donors per group. Bars are the mean±SD. *p<0.05, **p<0.01, ns, non-significant by Friedman one-way analysis of variance. CAR, chimeric antigen receptor; MCT, monocarboxylate transporters; NT, non-transduced; MFI, mean fluorescence intensity.

We first studied MCT-1 inhibition in combination with αCD19 CAR T cells expressing a 4-1BB co-stimulation domain (online supplemental figure 1A, B). MCT-1 (figure 1A) and its ancillary protein CD147 (figure 1B) were highly expressed on activated αCD19-CAR T cells compared with resting CAR and non-transduced (NT) T cells. MCT-4 staining was first validated by analyzing HEPG2 cells (MCT-4^+^), Raji (MCT-4^−^) and αCD19-CAR T cells (online supplemental figure 1C). MCT-4 had a similar high expression on NT, resting and activated αCD19-CAR T cells (figure 1C). In contrast, B-cell tumor cell lines express high levels of MCT-1 and CD147, but no expression of MCT-4 was detected (online supplemental figure 1D-F). To further explore the function of MCT-1 on CAR T cells, lactic acid accumulation was measured after being cultured with small molecules which specifically block MCT-1/2 at a nanomolar range; AZD3965 or AR-C155858. Raji cells quickly accumulated intracellular lactate after MCT-1 blockade with AZD3965 (figure 1D) or AR-C155858 (figure 1E), while activated αCD19-CAR T cells did not significantly accumulate lactic acid intracellularly. Similarly, MCT-1 inhibition decreased intracellular pH on Raji cells but not on activated αCD19-CAR T cells (figure 1F–G). Importantly, MCT-1 inhibition did not impact the expression of MCT-1/4 or CD147 on activated αCD19-CAR T cells (online supplemental figure 1G-I). MCT-1 blockade quickly induces apoptosis and diminishes the proliferation of tumor cells.^26^ However, no differences in CAR T-cell expansion were found by counting the number of effector cells after 4 or 7 days of exposure to MCT-1 blockers (figure 1H). Similarly, no significant difference was found in early apoptotic cells measured by annexin V staining on CAR T cells (figure 1I), while no significant increase was found in Raji cells (online supplemental figure 1J). These results suggest CAR T cells increase the expression of MCT-1 on activation, and pharmacology blockade of MCT-1/2 is not sufficient to significantly impair lactic acid export.

10.1136/jitc-2022-006287.supp1 Supplementary data

### MCT-1 blockade induces metabolic rewiring on CAR T cells

**Figure 2:**
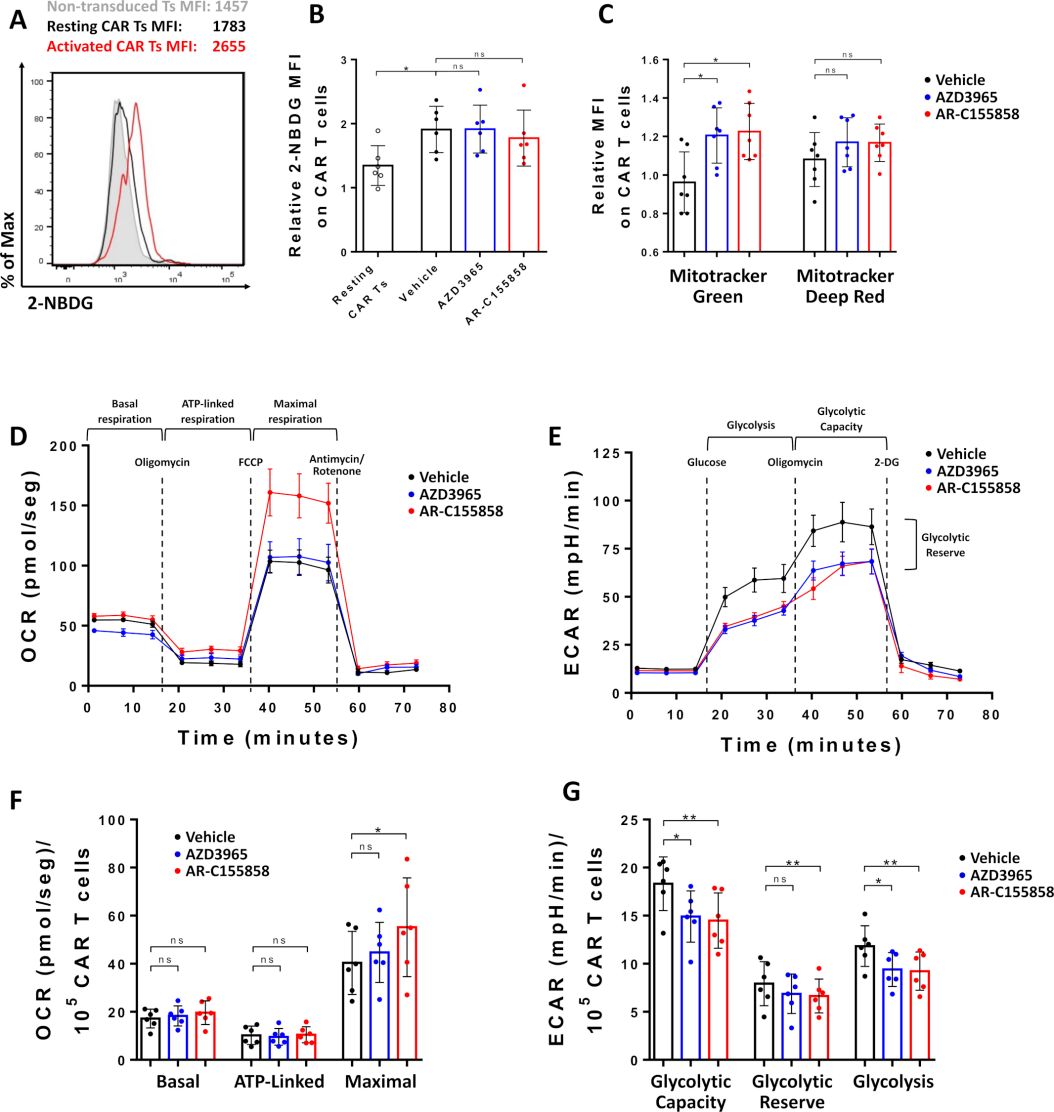
Metabolic characterization on CAR T cells on MCT-1 blockade. Non-transduced or αCD19-CAR T cells were cultured with Raji or Raji-CD19^KO^ and 100 nM of MCT-1 inhibitors for 24 hours. (A–B) Representative histogram and pooled data of 2-NBDG uptake. (C) Mitochondrial mass measured with Mito Tracker Green and Mito Tracker Deep Red staining. Pooled data of three independent experiments, n=7 healthy donors per group. Bars are the mean±SD. *p<0.05, ns, non-significant Friedman one-way analysis of variance (ANOVA). Representative plot of (D) oxygen consumption rate (OCR) and (E) extracellular acidification rate (ECAR) on CAR T cells activated for 24 hours with plate-bound αFMC63 antibody, n=7 replicates. Bars are the mean±SEM. (F) Basal, ATP-linked respiration and maximal respiration. (G) Glycolytic capacity, glycolytic reserve and glycolysis. Data from two independent experiments, n=6 healthy donors per group. Bars are the mean±SD. *p<0.05, **p<0.01, ns, non-significant by Friedman one-way ANOVA. CAR, chimeric antigen receptor; MCT, monocarboxylate transporters; MFI, mean fluorescence intensity; ATP, adenosine triphosphate; FCCP, Carbonyl cyanide-4 (trifluoromethoxy) phenylhydrazone.

We explored whether MCT-1 inhibition could rewire the metabolic network of CAR T cells, as has been observed in tumor models. Glucose uptake remained unchanged on αCD19-CAR T cells on MCT-1 blockade (figure 2A, B). In contrast, both AZD3965 and AR-C155858 increased mitochondrial mass on activated αCD19-CAR T cells (figure 2C), suggesting MCT-1 inhibition could induce metabolic reprogramming by increasing mitochondrial metabolism.

We next performed CAR T-cell metabolic profiling by real-time measurement of oxygen consumption rate (OCR) and extracellular acidification rate (ECAR). For these experiments, we used anti-CAR antibodies for activation to avoid background measurement from target cells (online supplemental figure 2). OCR and ECAR were measured on αCD19-CAR T cells activated overnight with plate-bound αFMC63 antibodies in the presence of MCT-1/2 blockers (figure 2D–E). MCT-1 inhibition with AZD3965 did not significantly increase basal, adenosine triphosphate (ATP)-linked or maximal respiration on CAR T cells, while AR- C155858 significantly increased maximal respiration on CAR T cells (figure 2F). Moreover, MCT-1 inhibition with both small molecules induced a partial reduction on the ECAR, reducing glycolysis and glycolytic capacity but not the glycolytic reserve values of CAR T cells (figure 2G). Importantly, incubation of MCT-1/2 blockers with Raji cells severely reduced their ECAR, indicating a higher sensitivity to MCT-1 blockade (online supplemental figure 3). Taken together, our results suggest MCT-1/2 blockade induced partial metabolic rewiring on CAR T cells.

10.1136/jitc-2022-006287.supp2 Supplementary data

10.1136/jitc-2022-006287.supp3 Supplementary data

### MCT-1 inhibition improved CAR T cells mediated cytotoxicity against B-cell lymphoma cell lines without impacting T-cell phenotype

**Figure 3:**
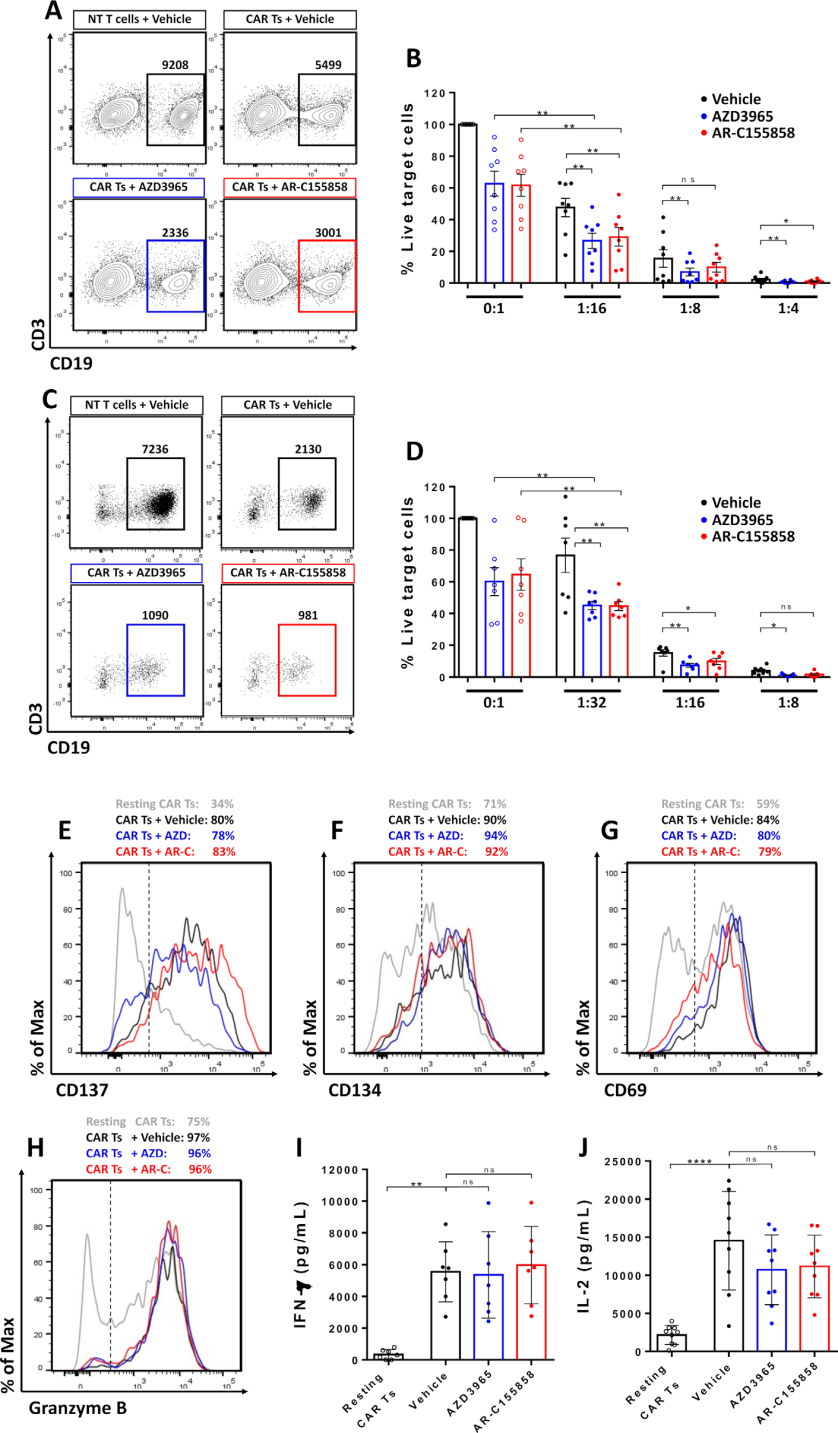
MCT-1 inhibition improved CAR T cells mediated cytotoxicity against B-cell lymphoma cell lines. MCT-1 inhibitors were added to non-transduced or αCD19-CAR T cells in culture with target cells for 48 hours. (A) Representative contour plot and count of Raji cells. (B) Percentage of live Raji cells cultured with CAR T cells at different effector:target ratios (E:T) ratios. (C) Representative contour plot and the total number of NALM-6 cells. (D) Percentage of live NALM-6 cells cultured with CAR T cells at different E:T ratios. Pooled data of three independent experiments, n=7–8 healthy donors per group. Bars are the mean±SEM. Expression of activation markers (E) 4-1BB, (F) OX40, (G) CD69 and (H) granzyme B on activated CAR T cells with Raji cells or Raji-CD19^KO^ for 24 hours. (I) IFN-γ and (G) IL-2 production was measured by ELISA after 48 hours. Pooled data of three independent experiments, n=7 healthy donors per group. Bars are the mean±SD. *p<0.05, **p<0.01, ***p<0.001, ****p<0.0001, ns, non-significant by Friedman one-way analysis of variance. CAR, chimeric antigen receptor; IFN, interferon; IL, interleukin; MCT, monocarboxylate transporters; NT, non-transduced.

Both CD19 CAR T cells and MCT-1 inhibition are active against B-cell lymphomas. We next tested the antitumor potential of combining CD19 CAR T cells with MCT-1 blockade. αCD19-CAR T cells were co-cultured with Raji and Raji CD19^KO^ cells on media with AZD3965 or AR-C155858 (figure 3A). Both inhibitors showed a 40% reduction of live tumor cells compared with vehicle control, while at 1:16 effector/target ratio (E:T), αCD19-CAR alone showed a 55% reduction of live tumor cells. In addition, the combination of MCT-1 blockers with CAR T cells increased the tumor cell killing compared with either treatment alone to 74% with AZD3965 and 71% with AR-C155858. Similar improvement in tumor killing was observed with higher E:T ratios (figure 3B).

The improved killing of CAR T cells against B-cell malignancies was also demonstrated against NALM-6, a widely used B-cell acute lymphoblastic leukemia cell line. Both AZD3965 and AR-C155858 showed a 30% reduction of live tumor cells compared with vehicle (figure 3C). At 1:32 (E:T) ratio, αCD19-CAR alone showed a 23% reduction of live tumor cells, while the combination improved the killing of tumor cells to 54% with both small molecules. Similar results were observed with higher E:T ratios (figure 3D).

### MCT1 blockade did not result in CAR T-cell exhaustion or differentiation

Metabolic networks are closely linked with T-cell memory formation and phenotype.^27^ Expression of 4-1BB (figure 3E), OX40 (figure 3F) and CD69 (figure 3G) were upregulated on CAR T cells on CD19-mediated activation with Raji cell lines but were not significantly altered after MCT-1 blockade with either AZD3965 or AR-C155858 (online supplemental figure 4A-C). Similarly, MCT-1 inhibition did not significantly reduce granzyme B expression on CD8 CAR T cells (figure 3H and online supplemental figure 3D) or interferon (IFN)-γ (figure 3I) and interleukin (IL)-2 (figure 3J) production on activated CAR T cells. Additionally, no differences in exhaustion markers PD-1 or T cell immunoglobulin and mucin domain-containing protein 3 (TIM-3) were found on CAR T cells (online supplemental figure 3E-K).

10.1136/jitc-2022-006287.supp4 Supplementary data

T cells memory formation is a node of integration of metabolic and phenotype as different memory T cells engage in distinct metabolic networks, including CAR T cells. To further explore if long-term MCT-1 blockade metabolic rewiring translates into phenotype changes, we analyzed the ex vivo T-cell memory phenotype. αCD19-CAR T cells were cultured for 21 days by weekly restimulation with Raji cells in the presence of MCT-1 blocking molecules. Different CAR T-cell memory populations were defined by CD45RA, CD45RO and CCR7 staining (online supplemental figure 5A, B). No significant differences were observed in the percentage of naïve T cells, effector memory T cells CD45RA, effector memory T cells or central memory T cells (online supplemental figure 5C-F) in the CAR T cells after 7, 14 and 21 days.

10.1136/jitc-2022-006287.supp5 Supplementary data

Different co-stimulatory domains within the CAR structure confer different metabolic programs, particularly 4-1BB stimulation favors oxidative metabolism while CD28 imprints a more glycolytic phenotype on T cells. To discard the effects observed were due to a particularity of 4-1BB signaling of the CAR structure, we performed similar experiments on the same CAR with a CD28 signaling domain instead of 4-1BB (online supplemental figure 6A). Similar cooperation on Raji cell killing was found combining CAR-CD28 CAR T cells with MCT-1 inhibition (online supplemental figure 6B), and no difference on 2-NBDG uptake, 4-1BB or CD69 expression, IFN-γ or IL-2 production was found after MCT-1 blockade (online supplemental figure 6C-G).

10.1136/jitc-2022-006287.supp6 Supplementary data

### Dual blockade of MCT-1 and MCT-4 severely impairs CAR T-cell effector functions

**Figure 4:**
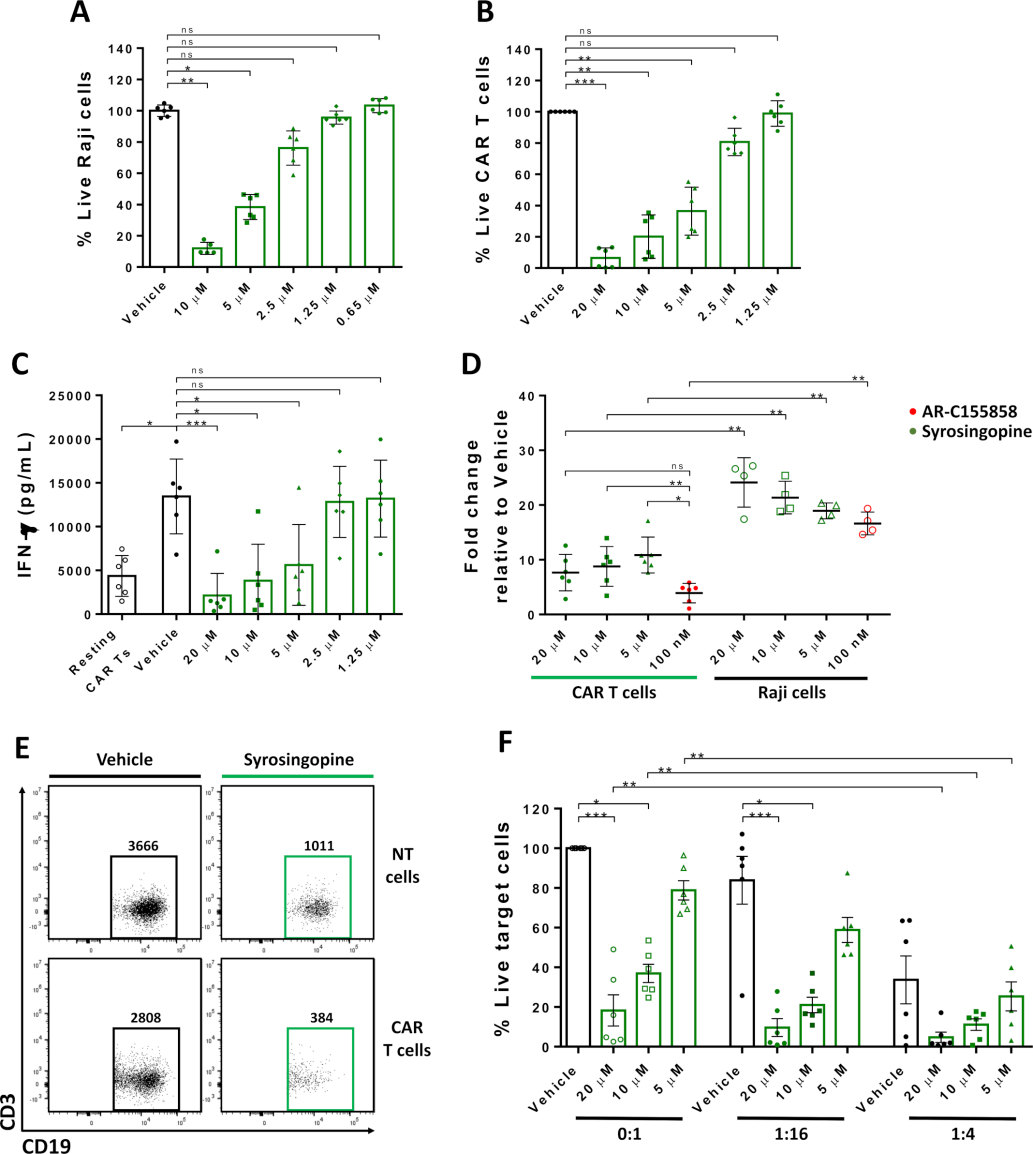
Dual blockade of monocarboxylate transporters-1/4 impairs CAR T-cell activation. Raji, non-transduced or αCD19-CAR T cells were cultured with different concentrations of syrosingopine or vehicle as control. (A) Percentage of live Raji cells or (B) αCD19-CAR T cells measured by flow cytometry. (C) IFN-γ production of activated CAR T cells was measured by ELISA after 48 hours. (D) Intracellular lactate relative to vehicle control on cells cultured with syrosingopine or AR-C155858. (E) Representative contour plot and the total number of Raji cells. (F) Percentage of live Raji cells cultured with CAR T cells at different effector:target ratios ratios. Pooled data of two independent experiments, n=6 healthy donors per group. Bars are the mean±SD. *p<0.05, **p<0.01, ***p<0.001, ns, non-significant by Friedman one-way analysis of variance. CAR, chimeric antigen receptor; IFN, interferon; NT, non-transduced.

We hypothesized that MCT-4 expression on CAR T cells is sufficient to exert lactate export in the absence of functional MCT-1. In contrast, dual inhibition of MCT-1 and MCT-4 might be expected to impair T-cell survival. To test this, αCD19-CAR T cells were cultured with syrosingopine, an anti-hypertensive drug which blocks lactate export through MCT-1 and MCT-4.^28^ Titration of syrosingopine showed sensitivity on Raji cells at the micromolar range (figure 4A), similar to previous works with other cell lines. In addition, the culture of activated αCD19-CAR T cells with syrosingopine significantly reduced CAR T-cell expansion (figure 4B) and IFN-γ production (figure 4C) in a dose-dependent manner. Moreover, significant lactate accumulation was found on syrosingopine-treated CAR T cells compared with MCT-1 inhibition with AR-C155858 (figure 4D), however, no reduction of MCT-1/4 was found on CAR T cells after culture with syrosingopine (online supplemental figure 7A, B).

10.1136/jitc-2022-006287.supp7 Supplementary data

We tested the potential cooperation of combining MCT-1/4 blockade with CAR T cells by measuring cytotoxicity against Raji cells on media with high doses of syrosingopine (figure 4E). Syrosingopine alone reduced the number of tumor cells but no statistical difference in tumor killing was found comparing CAR mediating killing with high doses of syrosingopine. Furthermore, increasing the CAR T-cell number improved tumor cell killing, but no difference was found between the combination and both treatments (figure 4F). Similarly, no differences on tumor cell killing were observed combining αCD19-CAR T cells and low doses of syrosingopine (online supplemental figure 7C). Taken together, our results indicate that dual MCT-1/4 blockade did not induce cooperation in killing tumor cells as observed with MCT-1 blockade (figure 3A–D). The substantial toxicity on CAR T cells and tumor cells after MCT-1/4 inhibition suggests CAR T cells function requires MCT-1 or MCT-4 for lactate export and dual inhibition severely impairs CAR T-cell antitumoral potential.

### The combination of MCT-1 blockade and CAR T cells improved efficacy in vivo

**Figure 5:**
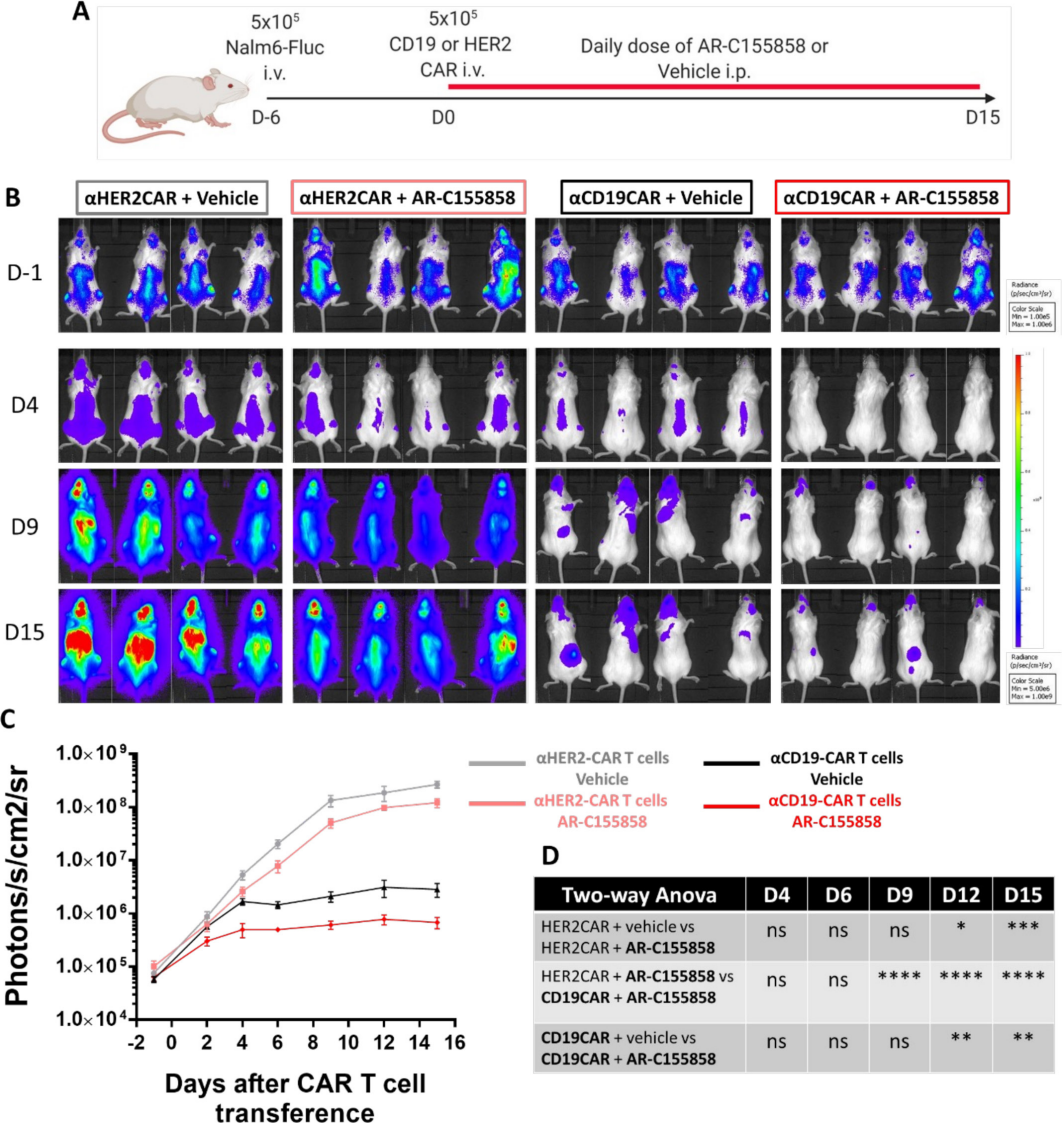
Combination of CAR T cells with monocarboxylate transporters -1 blockade improved T-cell antitumoral control against a B-cell leukemia xenograft animal model. 5×10^5^ NALM-6 cells expressing luciferase were intravenously inoculated on NOD/SCID mice. After 6 days, 5×10^5^ αCD19-CAR or αHER-CAR T cells were transferred intravenously, and intraperitoneal injections of AR-C155858 at 5 mg/kg were administered daily. (A) Overview of in vivo experiment. (B) Bioluminescence radiance (photons/s/cm^2^/sr) of NALM-6 tumors in NOD/SCID mice. (C) Geometric mean radiance of NALM-6 cells in all groups. (D) Table with statistical analysis through two-way analysis of variance (ANOVA) with multiple comparisons between groups at each time point. Bars are mean±SEM. *p<0.05, **p<0.01, ****p<0.001 ns, non-significant. Pooled data of two independent experiments, n=7–8. CAR, chimeric antigen receptor; i.p., intraperitoneal; i.v., intravenous.

The antitumoral potential of MCT-1 inhibition in combination with CAR T cells was tested in vivo. NALM-6/luciferase-burdened mice received CAR T cells, daily intraperitoneal injections of MCT-1 inhibitor, or both (figure 5A). AR-C155858 was used as more significant differences in CAR T-cell metabolic phenotype were found compared with AZD3965. An HER2 CAR was used as a negative control. MCT-1 blockade quickly reduced tumor burden 4 days after treatment (figure 5B), while αCD19-CAR T cells efficiently controlled tumor burden 9 days post transference. Moreover, the combination of MCT-1 and αCD19-CAR T cells significantly decreased tumor burden compared with either treatment alone (figure 5C, D).

**Figure 6:**
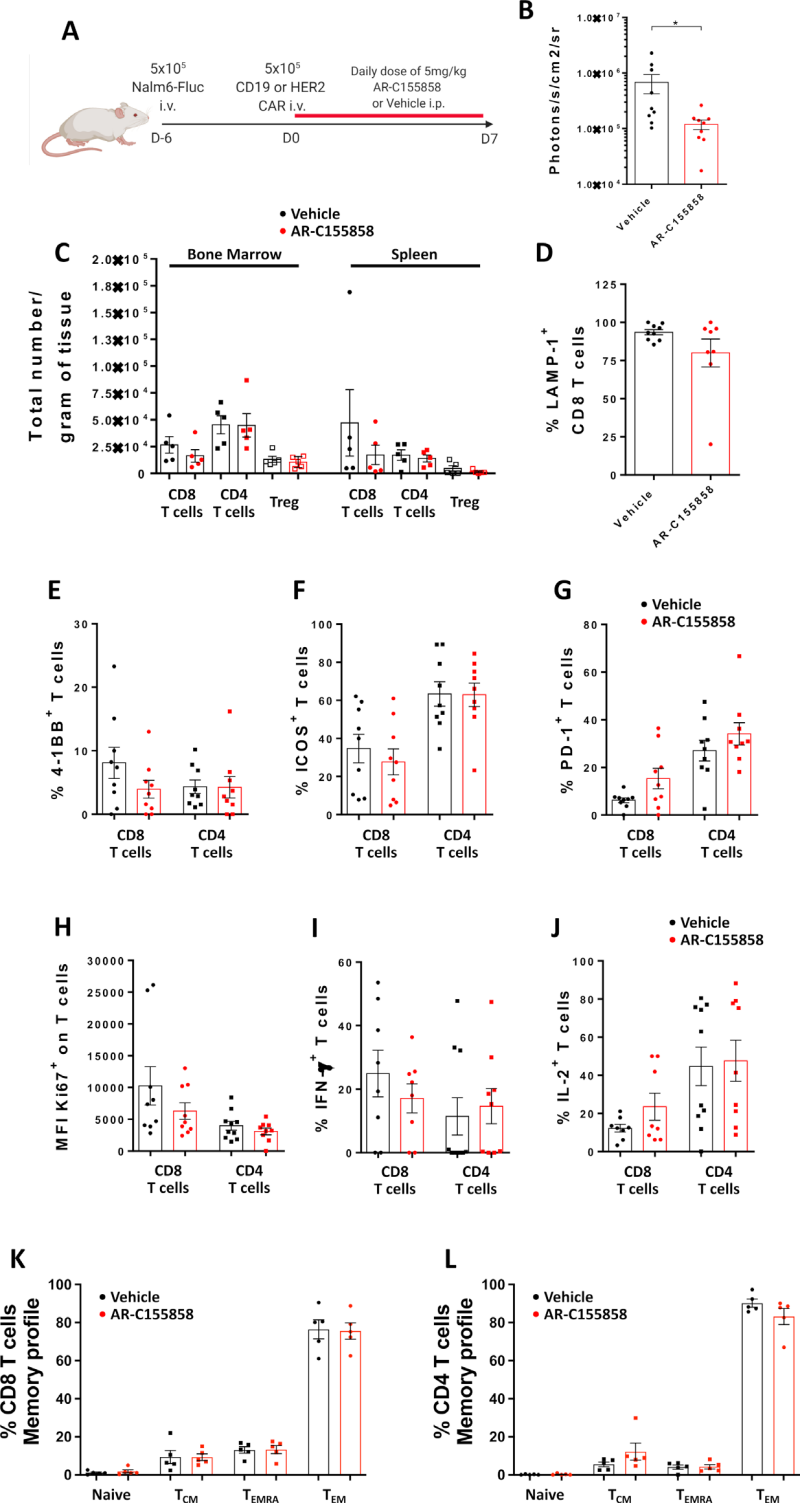
CAR T cells phenotype is unaffected after MCT-1 inhibition on a B-cell leukemia xenograft model. 5×10^5^ NALM-6 cells expressing luciferase were intravenously inoculated on NOD/SCID mice. After 6 days, 5×10^5^ αCD19-CAR were transferred intravenously and intraperitoneal injections of AR-C155858 were administered daily. After 7 days of treatment, bone marrow and spleen were analyzed by flow cytometry. (A) Overview of experiment. (B) Bioluminescence radiance (photons/s/cm^2^/sr) of NALM-6 tumors in NOD/SCID mice. (C) Total number of lymphoid cells on bone marrow and spleen. Representative data of two independent experiments, n=5 mice per group. Expression of (D) LAMP-1, (E) 4-1BB, (F) ICOS, (G) PD-1, (H) Ki-67, (I) IFN and (J) IL-2 on bone marrow-infiltrated T cells. Pooled data from two independent experiments, n=8–9 per group. Percentage of naïve T cells, central memory T cells (T_CM_), effector memory T cells CD45RA (TEMRA) and effector memory T cells (T_EM_) defined by expression of CCR7 and CD45RO on (K) CD8 and (L) CD4 T cells on bone marrow. Representative data of two independent experiments, n=5 mice per group. Bars are mean±SEM. *p<0.05, ns, non-significant by unpaired t-test with Welch correction. CAR, chimeric antigen receptor; ICOS, Inducible costimulator; PD-1, programmed cell death protein 1; IFN, interferon; IL, interleukin; i.p., intraperitoneal; i.v., intravenous; MFI, mean fluorescence intensity.

Given the different metabolic requirements between tumor cells and T cells cultured ex vivo and in vivo,^29^ we tested if long-term exposure to MCT-1 inhibitors impacts CAR T-cell phenotype in tumor-bearing mice. Phenotyping of bone marrow and splenic T cells was performed after 7 days of treatment with AR-C1585585 (figure 6A) in NALM-6 bearing mice transferred with αCD19-CAR T cells. The combination of αCD19-CAR T cells with MCT-1 significantly reduced the tumor burden after 7 days of treatment (figure 6B). No differences in the number of CD8 T cells, CD4 T cells or regulatory CD4 T cells in bone marrow or spleen after MCT-1 inhibition were found (figure 6C). Furthermore, no differences on LAMP-1 expression on bone marrow CD8 T cells were found (figure 6D). Similarly, 4-1BB, Inducible costimulator (ICOS), PD-1 or Ki67 expression (figure 6E–H) and IFN-γ and IL-2 production (figure 6I–J) were not affected by MCT-1 blockade on bone marrow infiltrating T cells. Consistent with our in vitro results, no differences in memory formation evaluated by CD45RO and CCR7 expression was found after MCT-1 blockade (figure 6K, L). Similar results were found on splenic T cells (online supplemental figure 8A-G), confirming that MCT-1 inhibition does not impact T-cell phenotype or functionality in vivo.

10.1136/jitc-2022-006287.supp8 Supplementary data

## Discussion

CAR T-cell therapy directed at B-lineage antigens is becoming established in the treatment of relapsed/refractory B-cell malignancies. However, while these therapies can induce sustained complete responses, most patients either do not enter remission or relapse. A proportion of CAR T-cell failure is caused by antigen escape, but most failure occurs in the presence of antigen. Notably, CAR T-cell failure is more likely to occur in the face of a high burden or rapidly progressing disease. For solid cancers is even worse. Infusion of tumor-infiltrating lymphocytes (TILs) or transgenic T-cell receptor therapies had clinical responses against melanoma, non-small cell lung cancer, among other solid tumors,^30 31^ but their efficacy is still limited, partially due to a metabolic hostile TME.^8^ There is a need to make ACT intrinsically more potent, or to combine them with synergistic therapeutics.

Modulating T cell or tumor/microenvironmental metabolism opens up a range of possibilities for enhancing CAR T-cell efficacy. For instance, increasing mitochondrial metabolism to produce a less-differentiated phenotype by manipulating the cultured media or adding IL-15 and IL-7 is a commonly used strategy.^32^ Additionally, combination of CAR T cells with metabolic regulators reducing glycolytic engagement, like AKT^33^ and mTOR^34^ have shown increasing antitumoral efficacy in preclinical models of B-cell leukemia and solid tumors. Similarly, directly regulating pyruvate fate by culturing αCD19 CAR T cells with a mitochondrial pyruvate carrier inhibitor dramatically improves CAR T cells efficacy in preclinical models of B-cell leukemia and solid tumors^35^ More recently, the identification of crucial amino acids depleted in the TME and genetic manipulation of CAR T cells to bypass nutrient scarcity has shown promising results in reshaping T-cell metabolism and improving their antitumoral potential.^36^ However, strategies which selectively affect tumor cells without hindering T-cell immunity have been challenging to develop, as tumor cells and proliferating T cells share many metabolic requirements.

Small molecules have been developed to exploit the Warburg effect. For instance, MCT are highly expressed across several cancers^37^ and small molecules which block lactate transporters such as MCT-1 and MCT-4 have been developed. These small molecules induce quick accumulation of lactic acid intracellularly, arresting proliferation and inducing cell death of tumor cells.^26^ Combining small molecules which exploit the Warburg effect with ACT is not straightforward however, since activated T cells increase glucose uptake and fermentation of glucose to lactate. Even so, as diffuse large B-cell lymphomas predominately expresses MCT-1 for lactate export, with little or no expression of MCT-4^24 25^ while T express both MCT-1 and MCT-4 on activation,^23^ we hypothesized that differential use of lactate transporters by tumor and T cells might allow selective metabolic disruption of tumor cells. More specifically, we sought to explore use of selective MCT-1 inhibitors for this strategy.

We found high expression of MCT-1, MCT-4 and CD147 on CAR T cells, with an increase in MCT-1 expression after CAR T-cell activation. MCT-1 inhibition increased mitochondrial mass, which correlated with increased OCR measurements on CAR T cells cultured with AR-C155858 while reducing their extracellular acidification rate but without impairing glucose uptake. Changes in lactate levels could induce metabolic rearrangement of CAR T cells without shutting down glycolysis while increasing the influx of metabolites towards the tricarboxylic acid cycle (TCA) cycle and mitochondrial biogenesis.^38^ However, we did not find any meaningful impact on CAR T cells phenotype, lactate accumulation, exhaustion, proliferation, or memory formation ex vivo, indicating the biological functions of CAR T cells were not significantly affected by MCT-1 inhibition. Limited information exists regarding functional differences between both MCT-1 inhibitors to account for the differences between AZD3965 and AR-C155858. Studies using mouse 4T-1 breast tumor cells found minor differences comparing both small molecules. AR-C155858 behaves like an MCT-1 substrate and with a trend toward higher uptake at lower pH with higher affinity to MCT-1 and MCT-2 compared with AZD3965, while the latter shows higher liposolubility, which explains its oral availability and different saturation rates compare with AR-C155858.^39^

Similar results were obtained in CAR T cells with a CD28 signaling domain, where no differences were observed in CAR T cells activation profile and cytotoxicity. However, we did not test a CD28 CAR T-cell in vivo or analyze its long-term activation. CD28 imprints a more glycolytic phenotype, with higher expression of GLUT-1 and higher acidification rates after 7 days activation,^11^ so it is possible different co-stimulation domains have different sensitivity to MCT-1 inhibition not addressed in this work. Optimizing CAR T cells signaling and their interaction with metabolic regulators could become a powerful tool for CAR T-cell therapy.

Our results suggest CAR T cells do not rely exclusively on MCT-1 for lactate export and other transporters could substitute this function, particularly MCT-4, which is expressed on activated T cells, including our CAR T cells. We tested this hypothesis by culturing CAR T cells with syrosingopine, a dual blocker of MCT-1 and MCT-4.^28^ Culture with syrosingopine impaired T-cell expansion, IFN-γ production and did not synergize with CAR T-cell killing of target cells, indicating CAR T cells rely at least on functional MCT-1 and MCT-4 for lactate export and T-cell functionality. These data are consistent with experiments on tumor models where MCT-4 expression is sufficient to reverse the effects of MCT-1 blockade.^40^ However, dual blocking of MCT-1 and MCT-4 with diclofenac showed minimal impact on T-cell activation and proliferation.^23^ Diclofenac has shown inhibition of lactate dehydrogenase activity and impaired glucose metabolism by inhibition of MYC on tumor models^41^; therefore, we opted to use syrosingopine in this study. Nevertheless, syrosingopine can bind to the glycolytic enzyme α-enolase, modulating glycolysis in cancer cell lines without impairing its enzymatic activity.^42^ Inhibiting α-enolase activity in CD8 T cells impairs their glycolytic and effector function,^43^ hence the role of syrosingopine in mediating T-cell immunity needs to be further studied.

The combination of CAR T cells with MCT-1 blockade improved tumor control in vitro on different B-cell malignancies models, NALM6 and Raji, implying the differential expression of MCTs on T cells and tumor cells offers a therapeutic opportunity. On a xenograph mouse model of B-cell leukemia, αCD19-CAR T cells efficiently reduced the tumor burden of NALM6 cells after 4 days, while daily injections of AR-C155858 intraperitoneally reduced tumor engraftment by around 50%. Furthermore, the combination of αCD19-CAR T cells and AR-C155858 significantly improved CAR T-cell antitumoral control after 12 days of treatment compared with each treatment alone without impairing T-cell phenotype on bone marrow and spleen infiltrated T cells. This work shows the potential of targeting metabolic susceptibilities of tumor cells by the cooperation of targeting lactate metabolism via MCT-1 in combination with a CAR T cells therapy against B-cell lymphoma. This approach may be broadened by exploration of MCT type expression different tumors, particularly subcategories of solid tumors expressing MCT-1 and negative for MCT-4, like melanoma, breast cancer, gliomas and others.^44 45^

The combination may have wider immune effects not explored in our model, especially in solid tumors. For example, tumor infiltrating regulatory T (Treg) cells which preferentially use lactate as a carbon source have been found to overexpress inhibitory molecules and increase their suppressive potential. Both tumour-infiltrating effector T cells and Treg cells can metabolize lactate, but only Treg cells have mechanisms to regenerate nicotinamide adenine dinucleotide (NAD)+ and avoid metabolic shutdown.^46 47^ Furthermore, pharmacological inhibition of MCT-1 or genetically editing this transporter on Treg cells increases the efficacy of αPD-1 treatment in mouse models.^20^ High lactic acid also impairs natural killer (NK) cells antitumoral potential^15^ and induces a tolerogenic phenotype on myeloid cells^48^ therefore, reduction of lactic acid production by MCT-1 inhibition could widen antitumoral immune responses. Additionally, our model is not well suited to studying the toxicities of this combination, including off-target effects of the small molecules. AZD3965 was tolerated in patients, but doses higher than 20 mg daily experienced dose-limiting side effects, with ophthalmic toxicity as the most common.^18^ Naïve T cells do not express MCT-1/2/4, and MCT-1 expression peaks first after 12 hours of activation, so it is possible systemic inhibition of MCT-1 causes disruptions in the initial steps of T-cell activation and proliferation, as shown in mouse CD8 T cells MCT-1^KO^.^49^ Furthermore, AZD3965 and AR-C155858 also block MCT-2, which is expressed in activated T cells and is not accounted for in this study. Developing and delivering monoclonal antibodies to block lactate transport through MCT-1 in the TME would reduce the risk of off-target effects and toxicities associated with the systemic administration of AZD3965.

Metabolic profile, tumor aggressiveness and immune cell phenotype are fundamentally linked, as further demonstrated by the metabolic shift in T cells after immune checkpoint therapy and the improvement of αPD-1 therapy after metabolic regulation.^50^ Detailed studies on immunocompetent mouse models infused with CAR T cells interrogating how changes in the tumor metabolic landscape after MCT inhibition and testing the combination in solid tumors models will lead to a deeper understanding of immune metabolism and better therapies against cancer.

In summary, pharmacological blockade of MCT-1 selectively impairs B-cell tumor growth without hindering CD19-specific CAR T-cell antitumoral potential, making the combination of both treatments an interesting approach against B-cell malignancies.

## Methods

### Cell lines and reagents

Iscove’s Modified Dulbecco’s Medium (IMDM) and Roswell Park Memorial Institute 1640 (RPMI Gibco—Life Technologies) were supplemented with 10% fetal bovine serum (Gibco) and 200 µM GlutaMAX (Gibco—Life Technologies). HEK 293T, HepG2, Raji and NALM-6 were obtained from the American Type Culture Collection (ATCC). Raji cell lines knockout of CD19 and NALM-6 expressing red luciferase were previously produced and validated.^51^ AZD3965, AR-C155858, and syrosingopine (MedChemExpres) were resuspended in dimethyl sulfoxide (DMSO) and stored at −80°C.

### Viral supernatant production by transient transfection

Retroviral particles were produced by transfecting with RD114, Peqpam-env and bicistronic plasmids containing RQR8 separated by 2A peptide and antigen-specific second-generation CARs. Transfection of HEK 293T was performed by seeding 1.8×10^6^ cells in 10 cm tissue culture plates over 24 hours. GeneJuice (Merck) was added to plain RPMI (30 μL to 470 μL of media) followed by incubation with the DNA mixture for 15 min. The DNA-GeneJuice mixture was added dropwise to the HEK 293T cells. Supernatants were harvested and pooled at 48 and 72 hours post-transfection.

### Isolation of mononuclear cells

Peripheral blood mononuclear cells (PBMCs) containing the lymphocytes fraction were isolated by Ficoll-Paque PLUS (GE HealthCare) separation. Blood from healthy donors was diluted to a 1:1 ratio with plain RPMI. The diluted blood was carefully layered onto the Ficoll-Paque solution. Each centrifuge tube was centrifuged at 750g for 40 min at 20°C with minimal acceleration and no brake. The buffy layer was harvested and washed twice with plain RPMI. NK cells depletion was performed by positive selection using CD56 MACS MicroBeads (Miltenyi Biotec) kit according to the manufacturer’s instructions. PBMC were counted, resuspended at 2×10^6^ cells/mL of complete RPMI and activated using CD3 (OKT3—0.5 µg/mL) and CD28 (28.2–0.5 µg/mL) antibodies (Miltenyi Biotec) and 100 IU/mL of IL-2 (Proleukin, Chiron) for 48 hours.

### PBMC transduction

Non-tissue treated 6-well plates were coated with 2 mL of phosphate buffered saline (PBS) containing RetroNectin (40 µg/mL—Takara) and incubated at 4°C overnight. PBS was aspirated from the plates and 3 mL per well of retroviral supernatant was added, incubated for 20 min at room temperature, followed by seeding of 2×10^6^ PBMC on RPMI supplemented with 400 IU/mL of IL-2. T cells were harvested 48 hours post-transduction and resuspended in fresh RPMI supplemented with 100 IU/mL of IL-2. CAR transduction efficiency was determined by staining RQR8 by fluorescence-activated cell sorting (FACS).

### FACS staining

Cells were counted and pelleted by centrifugation at 400g for 5 min in a 96-well culture plates, washed with 200 µL of PBS, resuspended in 100 µL of PBS containing fluorescent-conjugated antibodies and incubated for 30 min at 4°C in the dark. After incubation, the samples were washed once and resuspended in 200 µL for analysis. For intracellular staining, cells were incubated with 100 µL of Citofix/Cytoperm (BD Biosciences) for 20 min at room temperature and washed twice with Perm/Wash and stained according to the manufacturer’s instructions. MitoTracker Deep Red FM and MitoTracker Green FM (Thermo Fisher) staining were performed by incubating 50 nM of dyes on 100 µL of RPMI without serum at 37°C for 30 min. 2-NBDG (Thermo Fisher) labeling was performed by starving the cells for 30 min on 5% bovine serum albumin (BSA)-PBS followed by incubating 50 µM of dye on 100 µL of RPMI without serum at 37°C for 30 min. BD LSRFortessa Cell Analyzer (BD Biosciences) or CytoFLEX Flow Cytometer (Beckman Coulter) were used for data collection. Raw FACS data was analyzed using FlowJo V.10. The list of antibodies is summarized on online supplemental table 1.

10.1136/jitc-2022-006287.supp9 Supplementary data

### CAR T-cell activation and cytotoxicity assay

Activation assay was performed by culturing 5×10^4^ target cells and 5×10^4^ CAR T cells in 200 µL of RPMI media with small molecules or vehicle as negative control. Cells were incubated for 24–48 hours and stained for flow cytometry. Cytotoxicity assay was performed by culture of 2.5×10^4^ target cells in 200 µL of RPMI media with CAR T cells at different E:T ratios in the presence of small molecules or vehicle as negative control. Cells were incubated for 48 hours and stained for RQR8, CD19, CD3 and viability dye (eFlour 780) to distinguish target cells from T cells by flow cytometry. Absolute number of cells was calculated by adding 2 µL/well of CountBright Absolute Counting Beads (Invitrogen) according to the manufacturer’s instructions.

### Intracellular pH measurement

5×10^4^ CAR T cells were activated by culturing with Raji cell lines at a 1:1 ratio (E:T) for 48 hours in 200 µL of RPMI media with small molecules or vehicle as a negative control. Intracellular pH was measured using the Intracellular pH Calibration Buffer Kit (Invitrogen) according to the manufacturer’s instructions. Tumor and T cells were stained for RQR8, CD3 and viability dye to identify the CAR T-cell population. Cells were then washed with HEPES-based pH 7.4 buffer, labeled with pHrodo Green AM and PowerLoad concentrate and incubated at 37°C for 30 min. Cells were washed once with HEPES buffer, suspended in 100 µL of pH calibration buffer pH 6.5 with valinomycin/nigericin at 10 µM and incubated for 10 min. pHrodo Green AM fluorescence was measured by flow cytometry.

### Proliferation assay

Raji cells were incubated with 4 µg/mL mitomycin C for 2 hours at 37°C. The cells were washed five times with sterile PBS and 1×10^4^ target cells were cultured with 5×10^4^ CAR T cells (1:5 ratio) for 16 hours. For the initial absolute number of T cells (input), 5×10^4^ CAR T cells were counted by adding CountBright Absolute Counting Beads (Invitrogen) cells. Small molecules or vehicle was added to the culture and counted after 4–7 days, fold expansion was calculated by dividing the absolute number of CAR T cells by input.

### In vitro memory CAR T cells formation

5×10^5^ CAR T cells were activated by cultured with 5×10^4^ mitomycin C treated Raji cells in presence of 100 IU/mL of IL-2 RPMI media and 100 nM of MCT-1 inhibitors. Cells were counted and expanded after 4 days of culture by washing the media by spinning at 400 g for 5 min, discarding the supernatant and adding supplemented RPMI. At day 7, CAR T cells were counted and recultured with a new batch of 5×10^4^ mitomycin C treated Raji cells. This cycle was repeated at day 14 and 21 from the initial activation. Memory profile was assessed by staining of CD3, RQR8, CCR7, CD45RA and CD45RO and analyzed by flow cytometry.

### Measuring cytokine production by ELISA

Human IL-2 and IFN-γ were both quantified using the ELISA MAX kits (BioLegend) according to the manufacturer’s instructions. IL-2 and IFN-γ production were determined from clarified supernatant harvested from 2.5×10^4^ CAR T cells cultured with 2.5×10^4^ target cell lines for 48 hours.

### Lactate measurement

CAR T cells were cultured with Raji cell lines at a 1:10 ratio (E:T) for 24 hours. CAR T cells were purified by positive selection using the CD34 magnetic separation (Miltenyi) kit according to the manufacturer’s instructions. Intracellular lactate was measured by culture of 2×10^4^ CAR T cells or Raji cells with small molecules or vehicle as control for 6 hours and measure of luminescence by Lactate-Glo assay (Promega) according to the manufacturer’s instructions.

### Production αCD19 FMC63 anti-idiotype antibody

An antibody against the variable region of the αCD19 FMC63 present on the CAR was produced by transfection of ExpiCHO-S (Thermo Fisher Scientific) using plasmids coding for the heavy chain anti-FMC63-muIgG2a and the light chain anti-FMC63-muIgk (clone 136.20.1). ÄKTA start was used for protein purification with a HiTrap Protein G HP column according to the manufacturer instructions (GE HealthCare). The protein was dialysed with Slide-A-Lyzer Dialysis Cassettes (Thermo Fisher Scientific) in 1000× PBS at 4°C overnight. Quantification of protein was performed using Pierce BCA Protein Assay Kit (Thermo Fisher Scientific) according to the manufacturer’s instructions.

### Activation of CAR T cells with αCD19 anti-idiotype antibody

Incubation of 12 well non-treated culture plates were done with 500 µL of coating buffer (BioLegend) containing 5 µg/mL of αCD19 FMC63 antibody and stored overnight at 4°C. The media was carefully removed and 1 mL of 5% BSA-PBS blocking solution was added. After 20 min of incubation at room temperature, the blocking solution was removed and 4×10^6^ CAR T cells on 4 mL of RPMI supplemented with 100 IU/mL of IL-2 were added.

### Extracellular acidification rate and Oxygen consumption rate measurement

CAR T-cell ECAR and OCR were measured as previously described.^52^ XF cell culture microplate was coated with poly-D-lysine at 50 µg/mL (Sigma-Aldrich) for 24 hours at 4°C. 2×10^5^ cells per well were seeded on XF media supplemented with glucose, glutamine and pyruvate (Gibco) for 1 hour in a non-CO_2_ incubator. For OCR interrogation, 1 µM of oligomycin, 1.5 µM of Carbonyl cyanide-4 (trifluoromethoxy) phenylhydrazone (FCCP) and 1 µM of antimycin A were used (Agilent). ECAR interrogation was performed by adding 10 mM of glucose (Gibco), 1 µM of oligomycin (Agilent) and 50 mM of 2-DG (Sigma-Aldrich). ECAR and OCR were measured using Seahorse XF Analyzer (Agilent) according to the manufacturer’s instructions. For OCR parameters, non-mitochondrial respiration was subtracted. ATP-linked respiration was calculated as the mean OCR after oligomycin injection and maximal respiration as the mean OCR after FCCP injection. For ECAR parameters, non-glycolytic acidification was subtracted. Glycolysis was calculated as the mean ECAR after glucose injection and glycolytic capacity as the mean ECAR after oligomycin injection. Glycolytic reserve was calculated as the difference between glycolytic capacity and glycolysis.

### B-cell leukemia xenograft animal models

NOD.CB17-Prkdcscid/J (NOD/SCID) mice were bred and kept at the Kathleen Lonsdale Building animal facility of UCL. Eight-week-old, same sex mice were allocated randomly in groups of 4–5 per cage in the different experimental procedures. NALM-6 expressing luciferase (NALM6-Fluc) cells were inoculated by intravenous injection of 5×10^5^ cells. Six days after tumor inoculation, 5×10^5^ αCD19 FMC63 CAR or αHER2 CAR T cells were transferred intravenously on 200 µL of sterile PBS. AR-C155858 was administered by daily intraperitoneal injections of 5 mg/kg on 10% DMSO-PBS solution or vehicle as control. Tumor growth was monitored by injecting 200 µL of luciferase at 200 µg/mL (BioScience) intraperitonially and measuring luminescence (photons/s/cm^2^/sr) on the IVIS Spectrum In Vivo Imaging System (PerkinElmer).

In vivo T-cell phenotype was performed by obtaining femur and spleen from mice after 7 days of treatment with CAR T cells and small molecules. Spleen was mechanically dissociated and filtered through a cell strainer (70 µm—Falcon) into complete RPMI media. Cells were washed twice with sterile PBS and resuspended on ACK Lysing buffer (Gibco) for 5 min at room temperature and washed three times with RPMI. Bone marrow cells were obtained by chopping the tissue and incubating it on 5 mL of plain RPMI with 0.35 µg/mL Liberase (Roche) and 0.25 µg/mL DNAse (Sigma) for 30 min at 37°C. After incubation, 5 mL of RPMI with 5 µM of EDTA (Sigma) was added, filtered through a cell strainer (70 µm—Falcon) and washed twice with plain RPMI. Staining was performed by incubating the samples on 25 µL of human Fc block (Life Technologies) solution for 10 min at 4°C. The samples were washed and stained to FACS as previously described. For intranuclear staining, eBioscience Fixation/Perm diluent and Fixation/Permeabilization concentrate (Invitrogen) was used according to the manufacturer’s instructions.

BD FACSymphony Cell Analyzer (BD Biosciences) was used for data collection. Absolute numbers of cells per gram were calculated by CountBright Absolute Counting Beads (Invitrogen) and dividing the absolute number of cells by organ weight in grams. T cells were defined as human CD45^+^, mouse CD45^−^, CD3^+^ CD19^−^. Tregs were defined from the T cells population as CD4^+^, CD8^−^, CD25^+^, FOXP3^+^. This work was approved by the UK Home Office–approved project license PP3889251 and approved by the UCL Biological Services Ethical Review Committee. Animal Welfare Assurance number: F16-00014. UCL’s Bloomsbury AWERB establishment license number (X7069FDD2).

### Statistical analysis

Statistical analysis was performed using GraphPad Prism software (GraphPad Software). Paired t-tests were performed pairwise between relevant groups; when comparing more than two groups, a Friedman one-way analysis of variance was performed. For animal experiments, we used a sample size of >4 mice per group unless otherwise indicated. This is considered a standard sample size for these experiments and was sufficient to evaluate the effects of CAR T cells antitumor activity. Mice were randomized before the first tumor measurement. Investigators were not blinded to groups during experiments. Blinding was not possible due to the treatment schedule. Only one animal was excluded from the analysis due to lack of tumor engraftment detected pretreatment. A parametric unpaired t-test was performed between relevant groups. P value<0.05 was considered statistically significant; *p≤0.05, **p≤0.01, ***p≤0.001 and ****p≤0.0001.

## Data Availability

Data are available upon reasonable request.
