# Supplementary material for: Inhibition of lactate transport by MCT-1 blockade improves chimeric antigen receptor T-cell therapy against B-cell malignancies
Source: J Immunother Cancer. 2023 Jun 30;11(6):e006287. doi: 10.1136/jitc-2022-006287 (PMC10314680; doi:10.1136/jitc-2022-006287)
Supplement: Supplementary data [file jitc-2022-006287supp001.pdf]

[redacted]

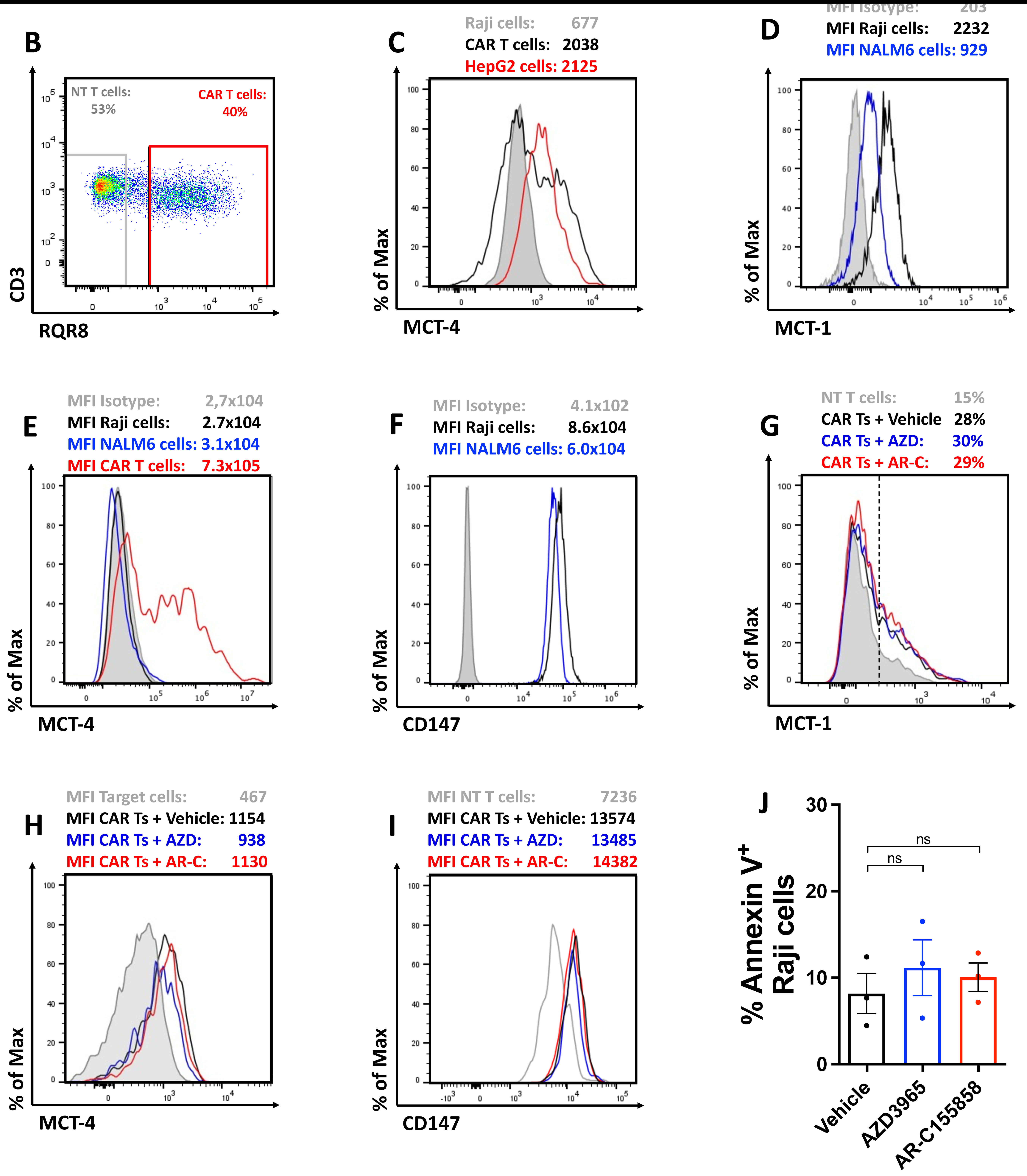

Supplementary figure 1: MCT expression on CAR T cells after MCT-1 pharmacological blockade. **(A)** Schematic of RQR8 and αCD19-FMC63-41BB CAR construct. **(B)** Representative plot of RQR8 expression on transduced T cells. **(C)** MCT-4 expression on Raji, CAR T and HepG2 cells. Expression of **(D)** MCT-1, **(E)** MCT-4 and **(F)** CD147 on Raji and NALM6 cells. Expression of **(G)** MCT-1, **(H)** MCT-4 and **(I)** CD147 on αCD19-CAR T cells cultured with target tumour cells and MCT-1 inhibitors for 24 hours, n=5-6 healthy donors per group. **(J)** Annexin V staining on Raji cells after 48 hours of culture with MCT-1 inhibitors, n=3. Data representative of two independent experiments.
