# Supplementary material for: Inhibition of lactate transport by MCT-1 blockade improves chimeric antigen receptor T-cell therapy against B-cell malignancies
Source: J Immunother Cancer. 2023 Jun 30;11(6):e006287. doi: 10.1136/jitc-2022-006287 (PMC10314680; doi:10.1136/jitc-2022-006287)
Supplement: Supplementary data [file jitc-2022-006287supp002.pdf]

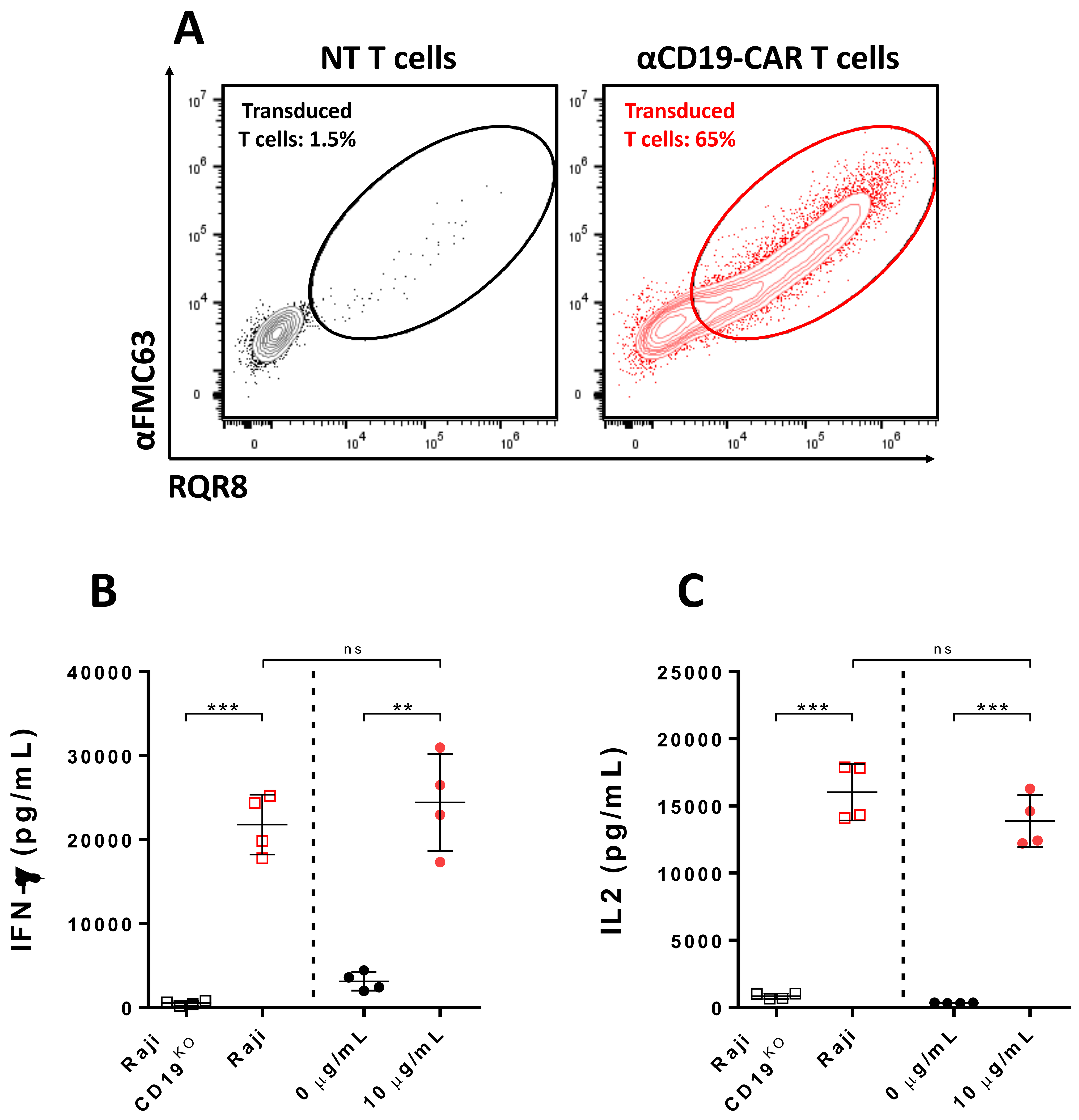

Supplementary figure 2: Activation of CAR T cells with plate-bound anti-idiotype antibody. (A) Representative histogram of αFMC63 antibody staining on non-transduced or αCD19-CAR T cells. **(B)** IFN-γ and **(C)** IL-2 production on CAR T cells activated with plate-bound αFMC63 or Raji cells for 24 hours. Pooled data of two independent experiments, n = 4 healthy donors per group. Bars are the mean ± SD. **p < 0.01, ***p < 0.001, ns = non-significant by Paired t-test.
