# Supplementary material for: Inhibition of lactate transport by MCT-1 blockade improves chimeric antigen receptor T-cell therapy against B-cell malignancies
Source: J Immunother Cancer. 2023 Jun 30;11(6):e006287. doi: 10.1136/jitc-2022-006287 (PMC10314680; doi:10.1136/jitc-2022-006287)
Supplement: Supplementary data [file jitc-2022-006287supp003.pdf]

BMJ Publishing Group Limited (BMJ) disclaims all liability and responsibility arising from any reliance placed on this supplemental material which has been supplied by the author(s)

[redacted]

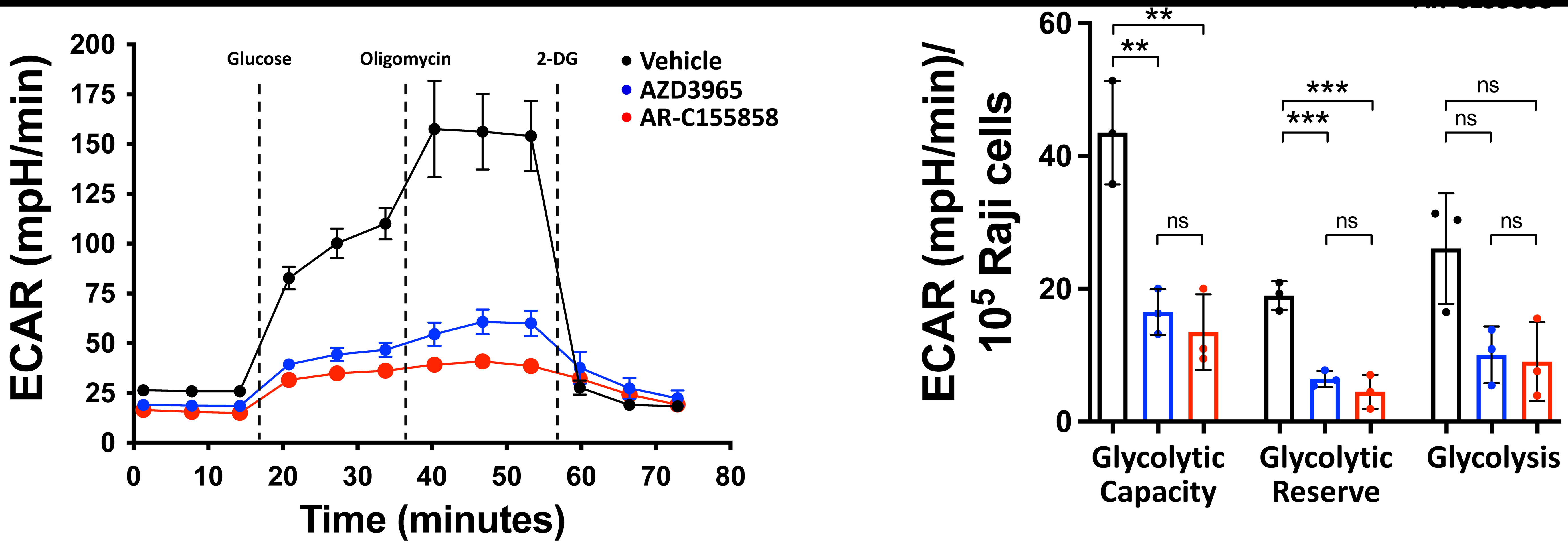

Supplementary figure 3: ECAR measurement on Raji cells after MCT-1 inhibition. (A) Representative plot of extracellular acidification rate (ECAR) on Raji cells cultured for 24 hours with MCT-1 inhibitors, n=5 replicates. Bars are the mean ± SEM. **(B)** Glycolytic capacity, glycolytic reserve and glycolysis. Data from three independent experiments. Bars are the mean ± SD. **p < 0.01, ***p < 0.001, ns = non-significant by Friedman One-Way ANOVA.
