# Supplementary material for: Inhibition of lactate transport by MCT-1 blockade improves chimeric antigen receptor T-cell therapy against B-cell malignancies
Source: J Immunother Cancer. 2023 Jun 30;11(6):e006287. doi: 10.1136/jitc-2022-006287 (PMC10314680; doi:10.1136/jitc-2022-006287)
Supplement: Supplementary data [file jitc-2022-006287supp004.pdf]

[redacted]

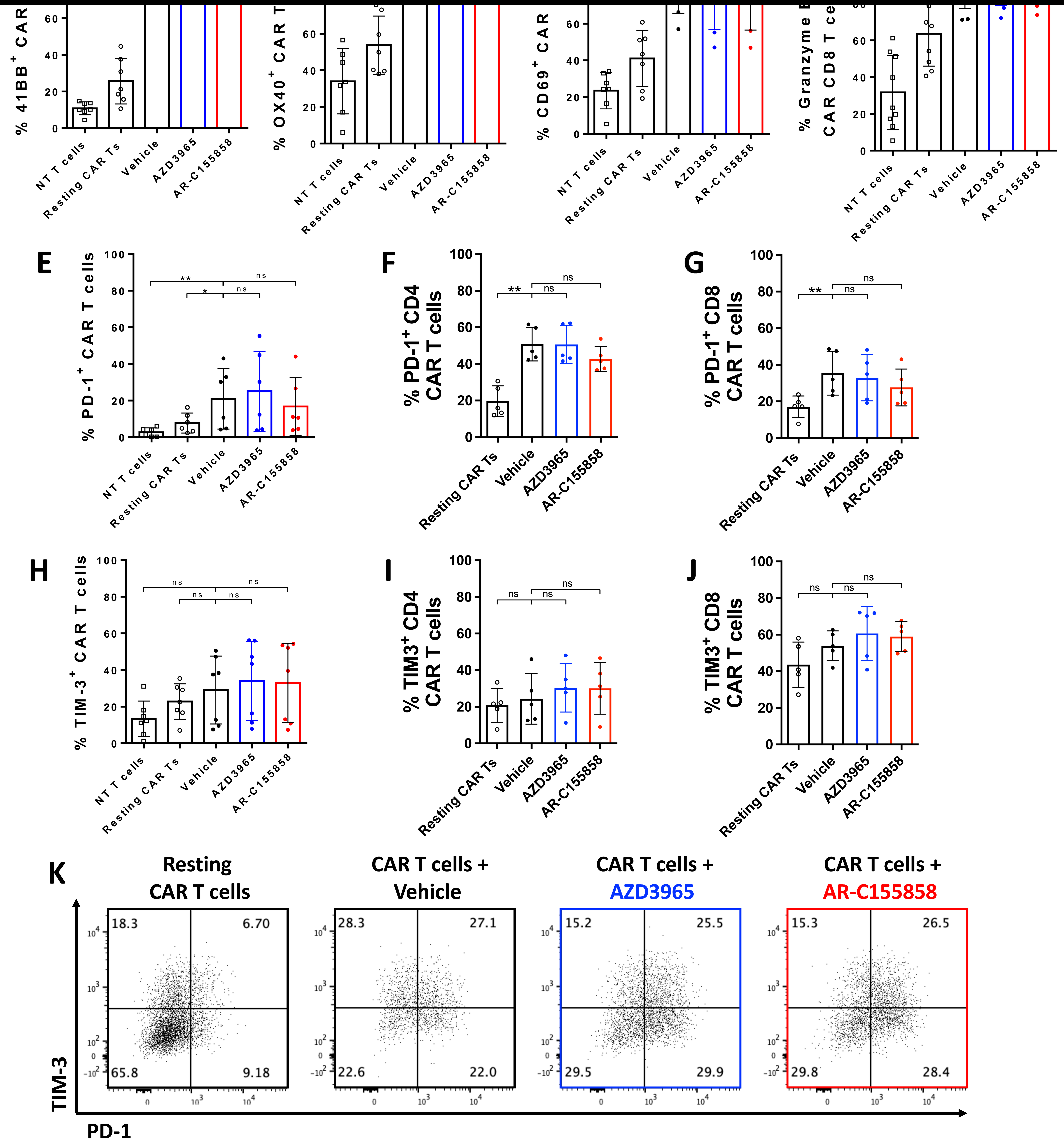

Supplementary figure 4: Expression of activation markers on CAR T cells after MCT-1 inhibition. Non-transduced or αCD19-CAR T cells were cultured with target tumour cells and MCT-1 inhibitors for 24 hours. Expression of (**A**) 4-1BB, **(B)** OX40, **(C)** CD69, **(D)** Granzyme B, (**E**) PD-1, (**F**) TIM-3 and (**G**) PD-1/TIM-3 on re-stimulated CAR T cells with Raji cells or Raji-CD19^{KO} (Resting CAR T cells) for 24 hours. Pooled data of three independent experiments, n = 7 healthy donors per group. Bars are the mean ± SD. *p < 0.05, **p < 0.01, ***p < 0.001, ns = non-significant by Friedman One-Way ANOVA.
