# Supplementary material for: Inhibition of lactate transport by MCT-1 blockade improves chimeric antigen receptor T-cell therapy against B-cell malignancies
Source: J Immunother Cancer. 2023 Jun 30;11(6):e006287. doi: 10.1136/jitc-2022-006287 (PMC10314680; doi:10.1136/jitc-2022-006287)
Supplement: Supplementary data [file jitc-2022-006287supp005.pdf]

BMJ Publishing Group Limited (BMJ) disclaims all liability and responsibility arising from any reliance placed on this supplemental material which has been supplied by the author(s)


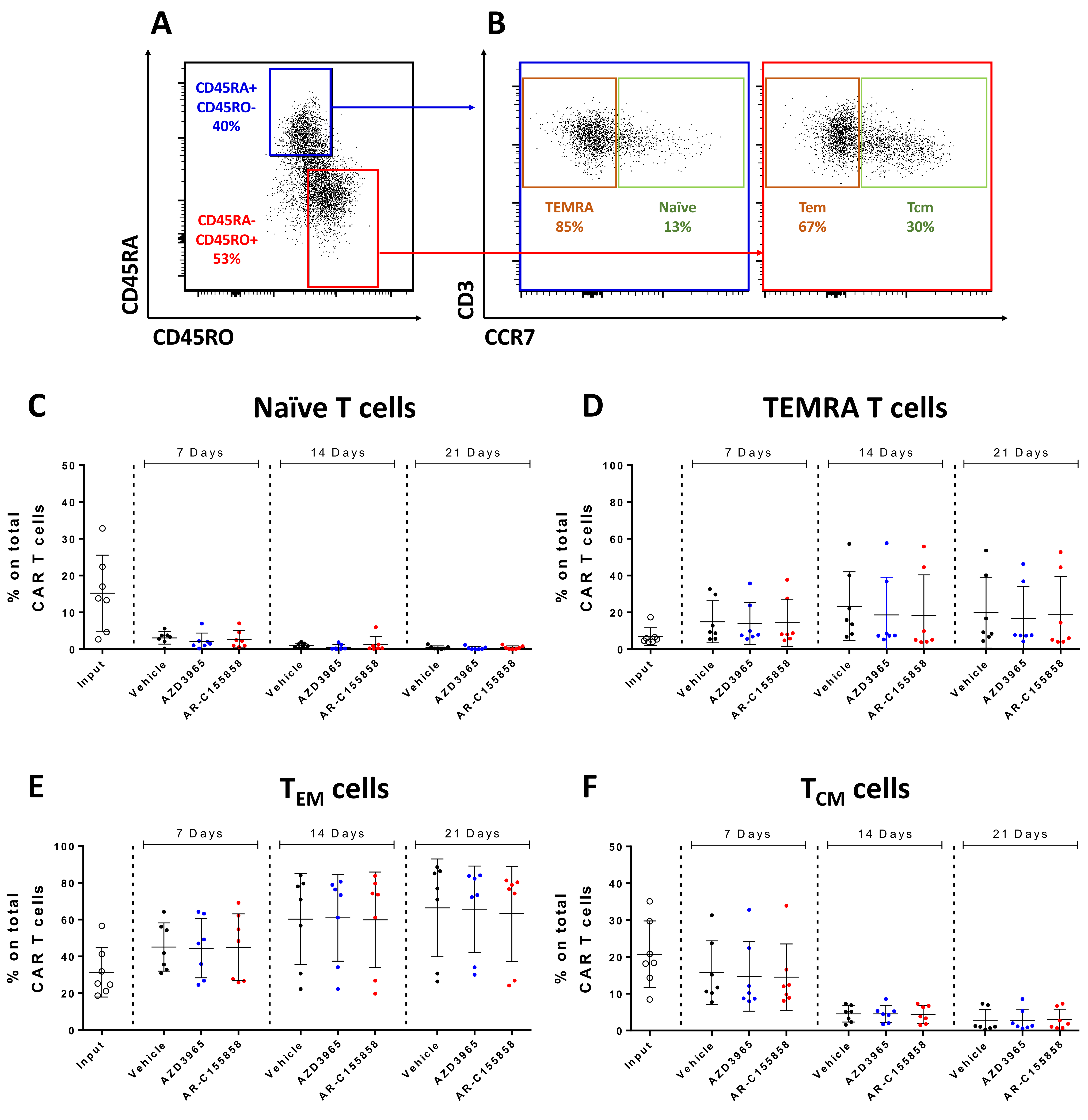


**Supplementary figure 5: Memory phenotype of CAR T cells after long exposures of MCT-1 blockers.** αCD19-CAR T cells were re-stimulated with Raji cells every 7 days. (**A-B**) Representative expression of CD45RA, CD45RO and CCR7 on CAR T cells. (**C**) Percentage of naïve T cells, (**D**) TEMRA T cells, (**E**) effector memory T (Tem) and (**F**) central memory T (Tcm) cells measured by flow cytometry every 7 days. Pooled data of three independent experiments, n = 7 healthy donors per group. Bars are the mean ± SD. ns = non-significant by Friedman One-Way ANOVA.
