# Supplementary material for: Inhibition of lactate transport by MCT-1 blockade improves chimeric antigen receptor T-cell therapy against B-cell malignancies
Source: J Immunother Cancer. 2023 Jun 30;11(6):e006287. doi: 10.1136/jitc-2022-006287 (PMC10314680; doi:10.1136/jitc-2022-006287)
Supplement: Supplementary data [file jitc-2022-006287supp006.pdf]

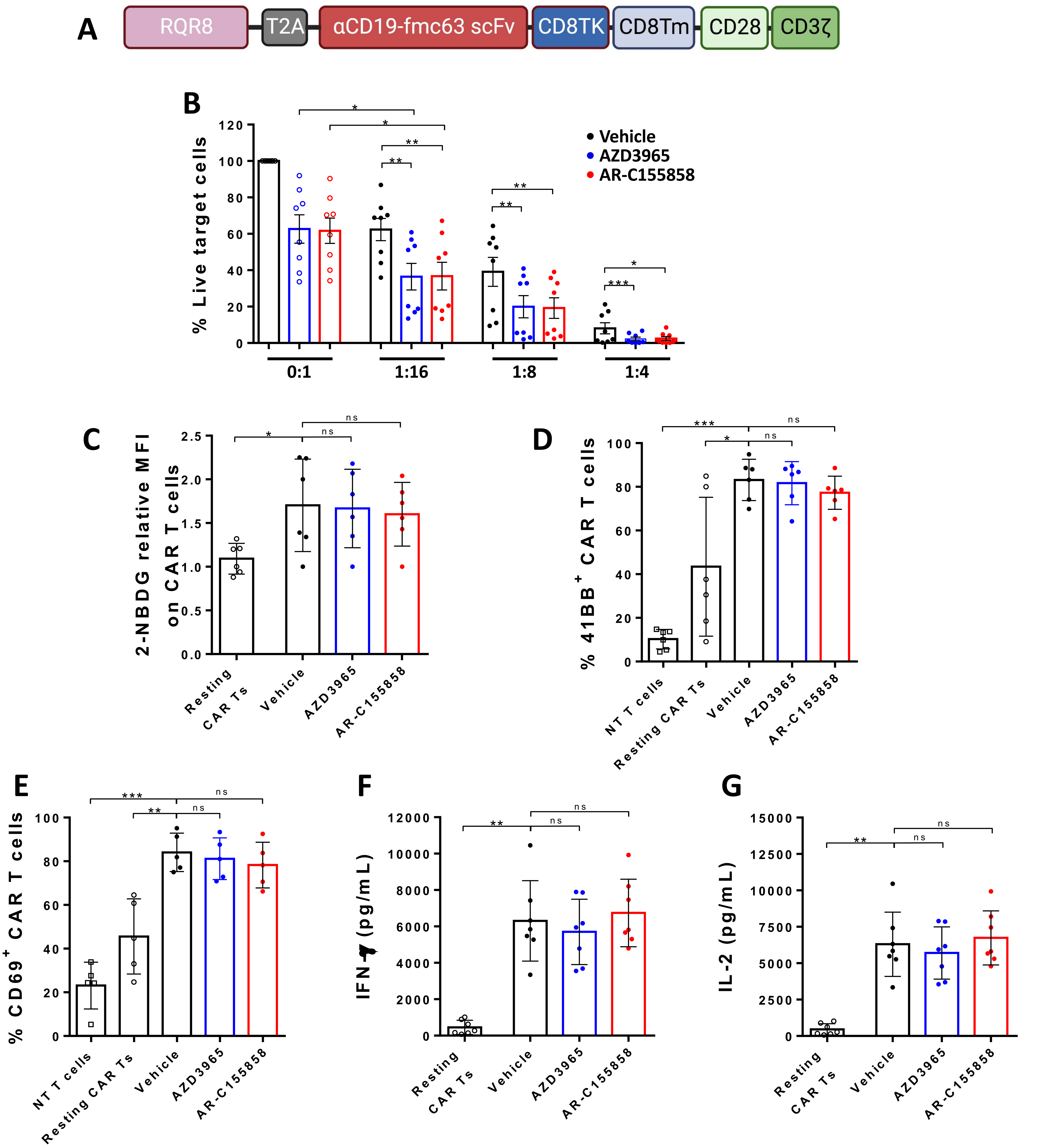

Supplementary figure 6: MCT-1 inhibition on CD19-CD28 CAR T cells. MCT-1 inhibitors were added to non-transduced or αCD19-CAR T cells with a CD28 co-stimulatory domain. **(A)** Schematic of RQR8 and αCD19-FMC63-CD28 CAR construct. **(B)** Percentage of live Raji cells cultured with CAR T cells at different effector: target ratios (E:T) ratios. **(C)** Relative MFI of 2-NBDG uptake. Expression of **(D)** 4-1BB and **(E)** CD69 re-stimulated CAR T cells with Raji cells or Raji-CD19^{KO} (Resting CAR Ts) for 24 hours. **(F)** IFN-γ and **(G)** IL-2 production measured by ELISA after 48 hours. Pooled data of two to three independent experiments, n=5-9 healthy donors per group. Bars are the mean ± SD. *p < 0.05, **p < 0.01, ***p < 0.001, ns=non-significant by Friedman One-Way ANOVA.
