# Supplementary material for: Inhibition of lactate transport by MCT-1 blockade improves chimeric antigen receptor T-cell therapy against B-cell malignancies
Source: J Immunother Cancer. 2023 Jun 30;11(6):e006287. doi: 10.1136/jitc-2022-006287 (PMC10314680; doi:10.1136/jitc-2022-006287)
Supplement: Supplementary data [file jitc-2022-006287supp007.pdf]

BMJ Publishing Group Limited (BMJ) disclaims all liability and responsibility arising from any reliance

placed on this supplemental material which has been supplied by the author(s)

[redacted]

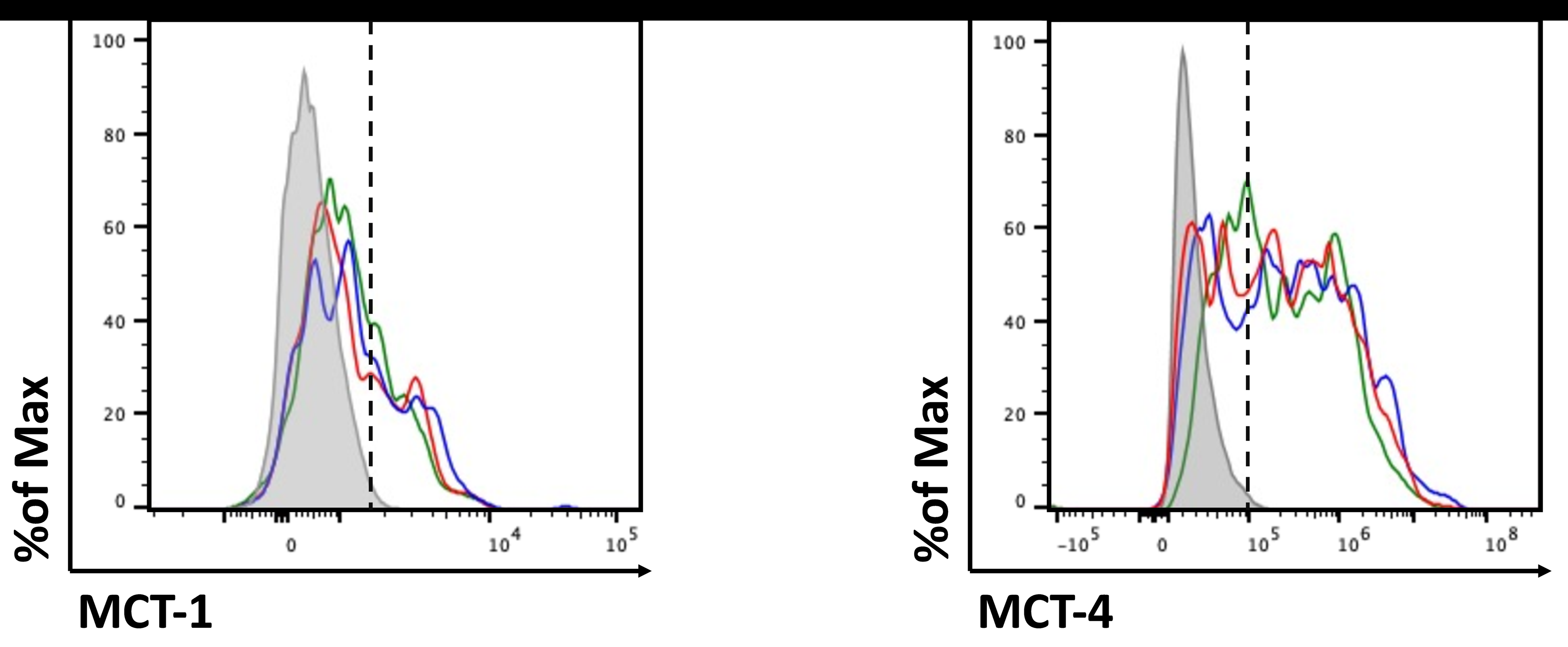

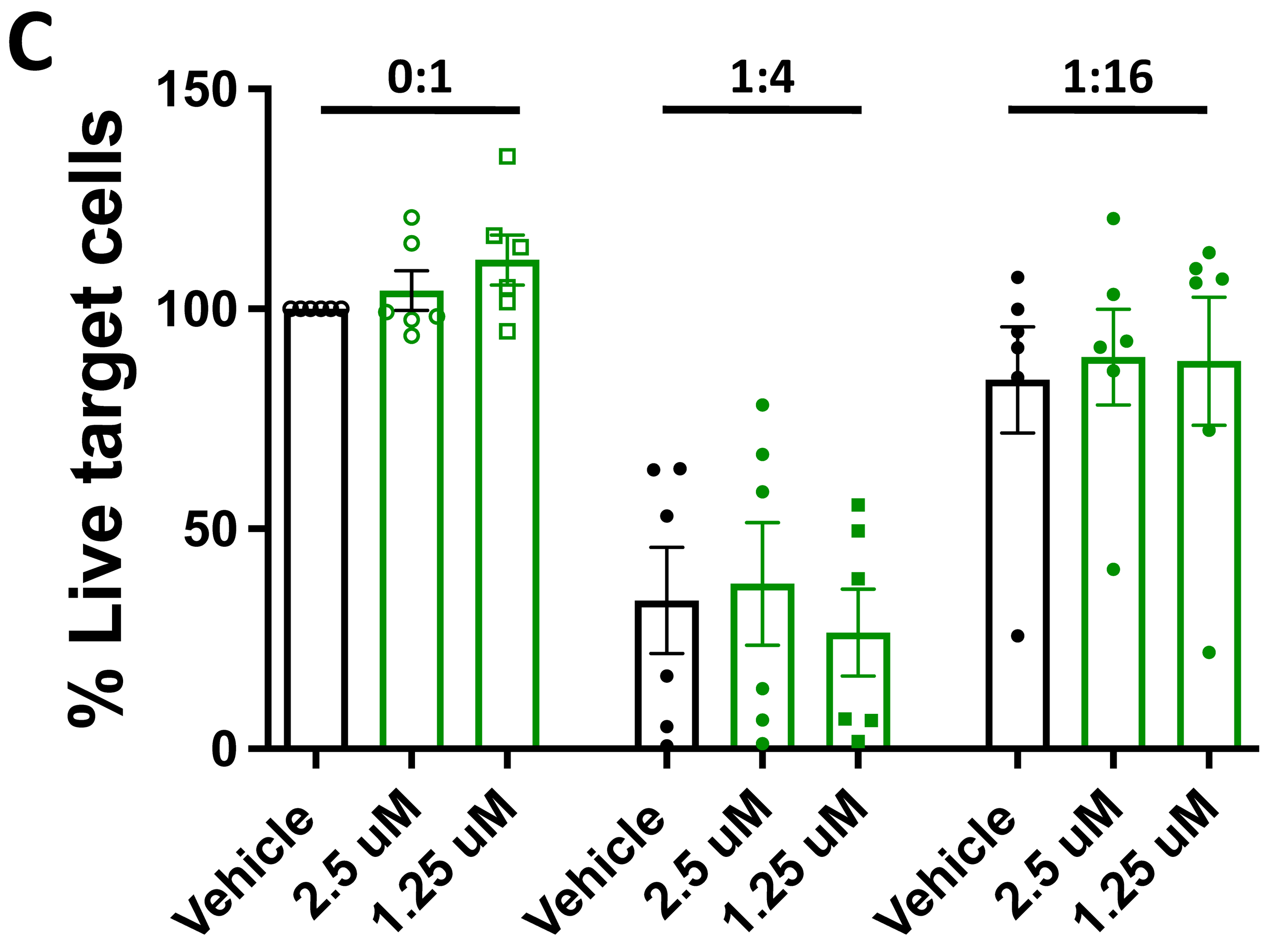

Supplementary figure 7: CAR T cells cultured with syrosingopine. αCD19-CAR T cells were cultured with different concentrations of syrosingopine or vehicle as control. Expression of **(A)** MCT-1 and **(B)** MCT-4 on αCD19-CAR T cells cultured with target tumour cells and syrosingopine for 24 hours. Representative data of two independent experiments, n = 4 healthy donors per group. (**C**) Percentage of live Raji cells cultured with CAR T cells at different effector: target ratios (E:T) ratios. Pooled data of two independent experiments, n = 6 healthy donors per group. Bars are the mean ± SEM.
