# Supplementary material for: Inhibition of lactate transport by MCT-1 blockade improves chimeric antigen receptor T-cell therapy against B-cell malignancies
Source: J Immunother Cancer. 2023 Jun 30;11(6):e006287. doi: 10.1136/jitc-2022-006287 (PMC10314680; doi:10.1136/jitc-2022-006287)
Supplement: Supplementary data [file jitc-2022-006287supp008.pdf]

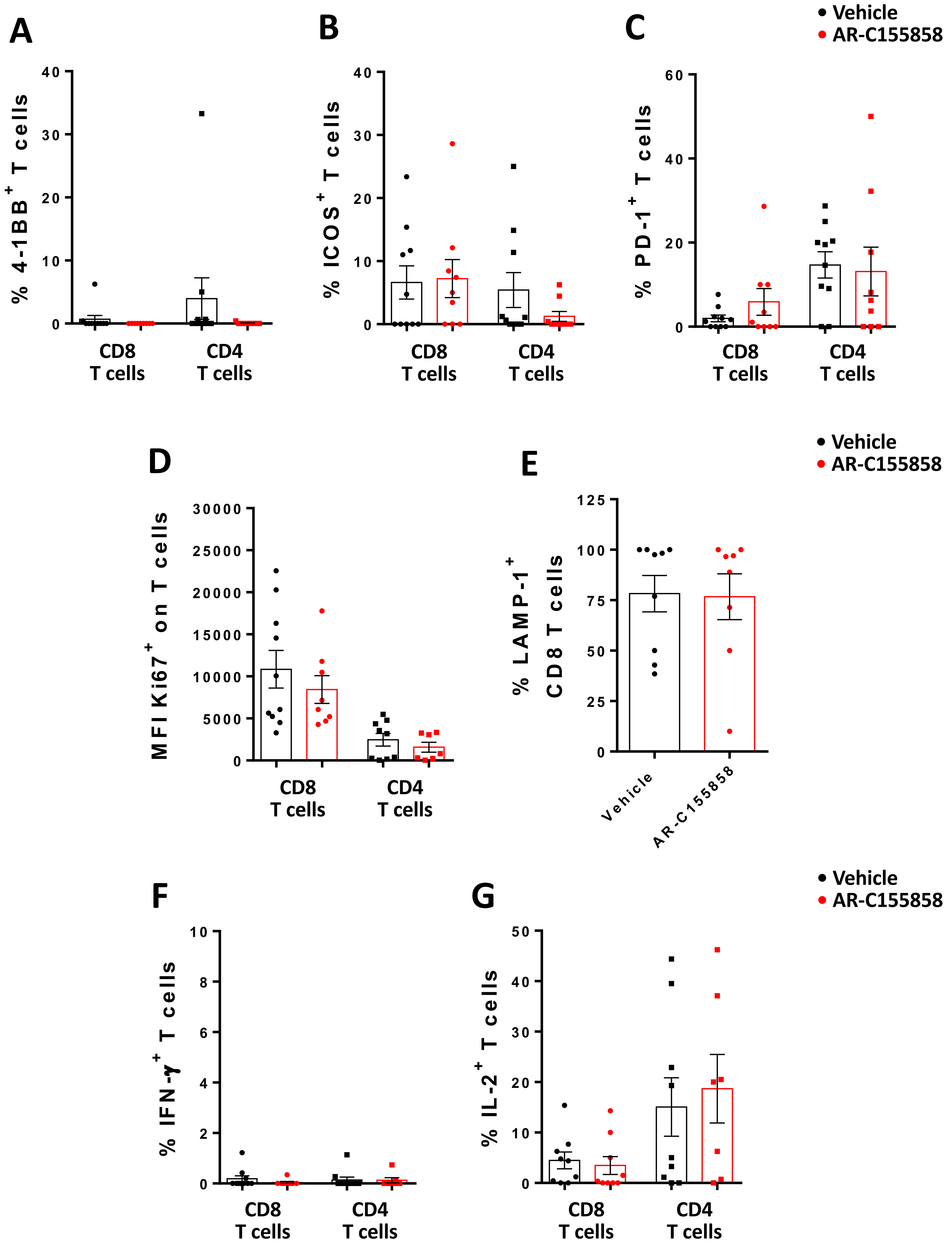

Supplementary figure 8: Spleen infiltrated CAR T cells phenotype after MCT-1 inhibition. Splenic T cells were analysed as described in figure 5. Expression of **(A)** 41BB, **(B)** ICOS, **(C)** PD-1, **(D)** Ki-67, **(E)** LAMP-1, **(F)** IFN and **(G)** IL-2 on splenic T cells. Pooled data from two independent experiments, n=8-9 per group. Bars are mean ± SEM. *p < 0.05, ns = non-significant by Unpaired t test with Welch correction.
