# Supplementary material for: Inhibition of lactate transport by MCT-1 blockade improves chimeric antigen receptor T-cell therapy against B-cell malignancies
Source: J Immunother Cancer. 2023 Jun 30;11(6):e006287. doi: 10.1136/jitc-2022-006287 (PMC10314680; doi:10.1136/jitc-2022-006287)
Supplement: Supplementary data [file jitc-2022-006287supp009.pdf]

Supplementary table 1: List of antibodies

REAGENT or RESOURCE	SOURCE	IDENTIFIER
**Antibodies**		
Anti-human CD19 FITC (SJ25C1)	Biolegend	363008
Anti-human CD366 FITC (F38-2E2)	Biolegend	345022
Anti-human CD3 FITC (HIT1a)	Biolegend	300306
Anti-human CD34 Alexa Fluor 488 (MAb11)	R&D Systems	FAB7227G
Anti-human CD147 Alexa Fluor 488 (MEM-M6/1)	Abcam	ab666
Anti-human CD197 PerCP/Cyanine5.5 (G043H7)	Biolegend	353219
Anti-human CD279 PerCP/Cyanine5.5 (EH12.2H7)	Biolegend	329914
Anti-human IFN-γ PE (4S.B3)	Biolegend	502509
Anti-human CD137 PE (4B4.1)	Biolegend	309804
Anti-human CD3 PE (HIT1a)	Biolegend	300308
Anti-human CD34 Alexa Fluor 488 (MAb11)	R&D Systems	FAB7227P
Anti-human CD137 PE/Dazzle 594 (4B4.1)	Biolegend	309826
Anti-human CD8 PE/Dazzle 594 (RPA-T8)	Biolegend	301058
Anti-human CD278 PE/Cy7 (C398.4A)	Biolegend	313520
Anti-human CD3 APC (HIT1a)	Biolegend	300312
Anti-human CD45RA APC (HI100)	Biolegend	304112
Anti-human CD19 APC (SJ25C1)	Biolegend	363006
Anti-human CD34 APC (MAb11)	R&D Systems	FAB7227A
Anti-human MCT1/SLC16A1 APC (MAb11)	R&D Systems	FAB8275A
Anti-human Granzyme B Alexa Fluor 647 (GB11)	Biolegend	515406
Anti-human Granzyme B Alexa Fluor 700 (QA16A02)	Biolegend	372222
Anti-human CD3 Pacific Blue (HIT1a)	Biolegend	300330
Anti-human CD45RO Brilliant Violet 421 (UCHL1)	Biolegend	304224
Anti-human CD366 Brilliant Violet 421 (F38-2E2)	Biolegend	345008
Anti-human FOXP3 Brilliant Violet 421 (206D)	Biolegend	320124
Anti-human CD25 Brilliant Violet 510 (M-A251)	Biolegend	356120
Anti-human CD2 Brilliant Violet 510 (RPA-2.10)	Biolegend	300218
Anti-human CD69 Brilliant Violet 605 (FN50)	Biolegend	310938
Anti-human IL-2 Brilliant Violet 650 (MQ1-17H12)	Biolegend	500334
Anti-human CD8 Brilliant Violet 650 (RPA-T8)	Biolegend	301042
Anti-human CD233 Brilliant Violet 650 (11C3C65)	Biolegend	369316
Anti-human CD107a Brilliant Violet 711 (H4A3)	Biolegend	328640
Anti-human CD134 Brilliant Violet 711 (Ber-ACT35)	Biolegend	350030
Anti-human CD4 Brilliant Violet 785 (OKT4)	Biolegend	317442
Anti-human Ki67 BUV395 (B56)	BD Bioscience	564071
Anti-human CD8 BUV496 (RPA-T8)	BD Bioscience	612943
Anti-human CD45RA BUV563 (HI100)	BD Bioscience	612926
Anti-mouse CD45 BUV661 (30-F11)	BD Bioscience	612975
Anti-human CD28 BUV737 (CD28.2)	BD Bioscience	612815
Anti-human CD3 BUV805 (SK7)	BD Bioscience	612894
Anti-human MCT4/ SLC16A3 (Polyclonal)	Proteintech	22787-1-AP
